# Therapeutic potential of isolated flavonoids from Anise and coriander aerial parts in antimicrobial efficacy, molecular docking, ADMET, and dynamic simulations

**DOI:** 10.1038/s41598-025-10927-w

**Published:** 2025-07-21

**Authors:** Salma El Sawi, Amal M. El-Feky, Mohamed Ibrahim El-Sayed, Ahmed F. El-Sayed

**Affiliations:** 1 Pharmacognosy Department, National Research Centre, 33El Buhouth St., Dokki, P.O. 12622, Giza, Egypt; 2 Department of Aromatic and Medicinal Plants, Pharmaceutical and Drug Industries Institute, National Research Centre, 33 El Buhouth St, P.O. 12622, Giza, Egypt; 3 Microbial Genetics Department, Biotechnology Research Institute, National Research Centre, Giza, Egypt; 4 Egypt Center for Research and Regenerative Medicine (ECRRM), Cairo, Egypt; 5 Molecular Modeling and Spectroscopy Laboratory, Centre of Excellence for Advanced Science, National Research Centre, Giza, Egypt

**Keywords:** *Pimpinella anisum* L., *Coriandrum sativum* L., Flavonoids, Anti-microbial activity, Docking, ADMET, Dynamic simulations, Biochemistry, Biotechnology, Computational biology and bioinformatics, Drug discovery, Microbiology, Plant sciences

## Abstract

Anise (*Pimpinella anisum*) and coriander (*Coriandrum sativum*) are globally esteemed for their applications in both culinary and medicinal contexts. In the present study, the quantitative analysis of total phenolic and flavonoid contents revealed that the aerial parts of anise contained elevated levels of phenolics (43.5 ± 0.23 mg/g) and flavonoids (39.8 ± 0.19 mg/g) in comparison to coriander, which exhibited values of 53.1 ± 0.18 mg/g for phenolics and 48.7 ± 0.21 mg/g for flavonoids. HPLC analysis revealed that quercetin was the most prevalent flavonoid, with chlorogenic acid and ellagic acid being the primary phenolic acids observed in both *P. anisum* and *C. sativum*. Eight flavonoids were isolated through column chromatography and silica gel thin-layer chromatography. The flavonoids identified from anise included isorhamnetin, kaempferol 3-*O*-rhamnoside, myricetin 3-*O*-rhamnoside, luteolin 7-*O*-β-D-glucopyranoside, and rutin, whereas coriander yielded quercetin, quercetrin, and kaempferol 3,7-*O*-dirhamnoside. Furthermore, the study highlighted the antimicrobial capabilities of anise and coriander extracts against pathogenic microorganisms, showing varying levels of effectiveness. Molecular docking was then utilized to examine interactions between promising compounds and antimicrobial target proteins. Results revealed that luteolin7-*O*-glucopyranoside, isorhamnetin, and quercetin exhibited strong binding energies, effectively binding to active sites of antimicrobial protein receptors. These interactions involved diverse molecular interactions, indicating the potential for enzyme inhibition and significant antimicrobial effects. Furthermore, in-silico ADMET profiles indicated alignment with Lipinski rules, suggesting advantageous physicochemical properties for these compounds. The MD simulations revealed stable complexes between luteolin7-*O*-glucopyranoside and antimicrobial receptors (1AD4, 2UV0, 2OV5, and 5TZ1). This stability was evidenced by RMSD values ranging from 0.18 to 0.40 nm, indicating minor fluctuations in RMSF values (0.10 to 0.60 nm). The SASA values ranged from 135 to 235 nm², and Rg values varied from 1.85 to 2.50 nm, offering insights into the protein complexes’ shapes. These results support the compounds’ potential in ongoing drug development efforts, highlighting their stability and suitability for further exploration in drug development processes.

**Supplementary Information:**

The online version contains supplementary material available at 10.1038/s41598-025-10927-w.

## Introduction

Aromatic herbs play a crucial role in global culinary practices, enhancing dish flavors and prolonging shelf life due to their sensory qualities and preservative characteristics^1^. Notably, anise (*Pimpinella anisum* L.) and coriander (*Coriandrum sativum* L.), both from the Apiaceae family, are extensively used for their culinary and medicinal advantages. These herbs offer numerous health benefits, such as aiding digestion, alleviating spasms, providing diuretic effects, and exhibiting anti-inflammatory properties^2,3^.

Anise, which is indigenous to the Mediterranean region, has been widely utilized in culinary practices and traditional medicine^4^. Its bioactive constituents have played a significant role in addressing ailments such as nightmares and depression^5^. From a chemical perspective, anise is composed of proteins, lipids—primarily fatty acids including palmitic and oleic acids—carbohydrates, and volatile oils, with trans-anethole being the most abundant component^6,7^. Although research has explored the phenolic content of anise roots^8^, there is a scarcity of comprehensive information regarding the aerial parts. Additionally, variations in chemical composition can arise from factors such as the area of origin, climatic conditions, soil characteristics, and the protocols utilized^9^.

Coriander, cultivated across Europe, Africa, and Asia, is valued for its distinctive fragrance and diverse applications of the leaves and seeds in cooking, cosmetics, and traditional medicine^10^^,^^11^. Rich in polyphenolic compounds, coriander leaves and fruits exhibits notable antioxidant, antidiabetic, and antimicrobial properties^12^^,^^13^. Consequently, these compounds may serve as viable alternatives for food preservation^14^. The beneficial properties are attributed to the presence of carotenoids, tannins, phenolic acids, flavonoids, and terpenes^10^^,^^15^, alongside significant levels of vitamins B12, C, and A^16^. The roots and seeds of coriander are rich in bioactive phytochemicals, such as gallic acid, thymol, and bornyl acetate. While linalool—the dominant volatile compound—plays a crucial role in its therapeutic effects. The composition and yield of coriander’s bioactive components vary based on genetic, environmental, and processing factors^17^.

Consequently, this research primarily aims to examine the composition of phenolics and flavonoids in the aerial parts of anise and coriander, as well as to isolate and structurally characterize the key flavonoids present. Additionally, it evaluates the antimicrobial efficacy of these plants against various microorganisms, including gram-negative and gram-positive bacteria, alongside *Candida albicans*. To further elucidate the relationship between these phytochemicals and their antimicrobial activity, computational approaches, particularly molecular docking, are employed. As molecular docking predicts the orientation of small therapeutic compounds within protein targets, it enables the assessment of affinity and biological activity, serving as a cornerstone in rational drug design. Given its significance in biomedical research, ongoing advancements in docking algorithms aim to enhance prediction accuracy^18^. Moreover, docking studies provide insights into compound–protein interactions, revealing binding configurations and potential therapeutic applications^19^^,^^20^^,^^21^.

## Materials and methods

### Chemicals, reagents and experiments

All the chemicals and solvents, used in the study were of high analytical quality. The phenolic and flavonoid standards utilized for HPLC analysis were acquired from Sigma-Aldrich Co., USA. For the purpose of analytical thin-layer chromatography (TLC), silica gel plates containing a fluorescent indicator 254 nm were employed. These plates were made of aluminum sheets 20 × 20, with a layer thickness of 0.2 mm and obtained from Merck. In the case of column chromatography, glass columns of different sizes were utilized. These columns were packed with silica gel G60 for the purpose of chromatographical adsorption analysis. The silica gel was sourced from BDH in England.

To determine the melting points, Koffler’s heating stage microscope was utilized. Mass spectra were obtained using the Finnigan Model 3200 Mass Spectrometer at 70 eV. Nuclear magnetic resonance spectra (NMR) were recorded using the JEOL EX-500 MHz Nuclear Magnetic Resonance spectrometer for the determination of ^1^H-NMR, and the 125 MHz spectrometer for the determination of ^13^C-NMR.

### Plant materials

#### Collection and identification

Fresh anise (*Pimpinella anisum* L.) and coriander (*Coriandrum sativum* L.) aerial parts were collected in March 2024 from private farm in Faiyum Governorate. The identification of these fresh plant specimens was kindly performed by Mrs. Trease Labib. who serves as the head consultant for plant identification at the Agricultural Ministry, located in the Orman Botanical Garden, Giza, Egypt. The collected aerial parts were arranged on sheets of non-glossy paper and air-dried in a shaded environment with sufficient air circulation for several days until they reached complete dryness and were subsequently ground into a fine powder. A specimen was then deposited in the herbarium of the National Research Centre (NRC) in Cairo, Egypt, with Voucher No. M269 and M270.

#### Extraction

The aerial parts of anise and coriander, each in powdered form and weighing 200 g, were defatted using petroleum ether (2 L, five times). After defatting, the powders were extracted with methanol (2 L, seven times) as a polar solvent for the extraction of flavonoids, following the standard cold maceration procedure^22^. The filtrates obtained were concentrated separately via rotary evaporation (Heidolph, Germany) at a temperature of 50° C, yielding 46% for anise and 56% for coriander. The concentrated extracts were subsequently stored in a refrigerator for chemical and biological investigations.

### Phytochemical investigation

#### Flavonoids screening

The methanol extracts of the aerial parts of *Pimpinella anisum* and *Coriandrum sativum* were systematically screened for flavonoids using both chemical reaction techniques and chromatographic methods to ensure comprehensive identification and characterization. Initially, the presence of flavonoids was assessed through two chemical tests. The **Doloking et al.**^23^ method utilized concentrated hydrochloric acid (HCl) and magnesium, triggering a characteristic color change indicative of flavones and flavonols. Additionally, **Ukoha et al.**^24^ test involved the application of diluted ammonia solution and 1% aluminum chloride (AlCl₃), where the formation of a yellow color confirmed the presence of flavonoid compounds, particularly those belonging to the flavone and flavonol subclasses.

To further determine the structural properties of the flavonoids, thin-layer chromatography (TLC) was employed following the protocol established by^25^. The chromatography process used a mobile phase consisting of ethyl acetate, formic acid, acetic acid, and water in a precise ratio of 100:11:11:26 (v/v/v/v), ensuring optimal separation of flavonoid compounds. After development, the chromatographic plates were sprayed with AlCl₃ reagent, a well-known fluorophore that enhances flavonoid visibility under ultraviolet (UV) light^26^. This step allowed for the observation of fluorescent bands specific to flavonoids, aiding in their identification and differentiation. The combination of chemical screening and chromatographic separation ensured reliable detection and classification of flavonoid constituents present in the methanol extracts.

#### Phenolics and flavonoids quantification

The assessment of total phenolic content was conducted using the Folin-Ciocalteu reagent, with the findings reported in terms of gallic acid equivalents (mg/g gallic acid equivalent)^27^. In addition, the total flavonoid content was evaluated as per the guidelines provided by **Zilic et al.**^28^ and expressed as catechin equivalents (mg/g of catechin equivalent).

#### Flavonoids identification

HPLC analysis was performed using an Agilent 1260 series to identify the phenolic acids and flavonoids present in the methanol extract of the aerial parts of both *P. anisum* and *C. sativum*. The separation was carried out using Zorbax Eclipse Plus C8 column (4.6 mm x 250 mm i.d., 5 μm). The parameters of the analysis were established based on the guidelines provided by **Hamed et al.**^29^.

#### Isolation of major flavonoids

The methanol extract of the aerial parts of both *P. anisum* and *C. sativum* (5 g) was chromatographed over a silica gel column (100 × 8 cm), using a mixed solvent of CH_3_Cl and MeOH in varying ratios (90:10, 70:30, 50:50, 30:70, 10:90) to afford 5 subfractions (SF1–SF5). ***For anise aerial parts***, subfractions 1 and 2 were further purified on a TLC silica gel plate using a developing system of CH_3_Cl and MeOH (4:1) to yield compounds 1 (26 mg) and 2 (19 mg), respectively. Subfraction 3 was purified on a column chromatography over a silica gel column (60 × 4 cm) using a solvent of ethyl acetate: ethanol (9.8: 0.2), resulting in the isolation of compound 3. Subfractions 4 and 5 were purified on a TLC silica gel plate using a developing system of CH_3_Cl and MeOH (4:1) to yield compounds 4 and 5, respectively. Similarly, ***for coriander aerial parts***, subfractions 1, 2, and 3 were separately chromatographed over silica gel TLC with CH_3_Cl and MeOH (4:1) to obtain compounds 6 (28 mg), 7 (31 mg), and 8 (19 mg), respectively. Upon exposure to NH_3_ vapors, the isolated compounds underwent a transformation from purple to yellow, thereby signifying the presence of flavonoid nature. The characterization and identification of these isolated flavonoids were accomplished through the utilization of spectroscopic techniques, namely Mass Spectrometer (Finnigan Model 3200 Mass spectrometer at 70 eV) and NMR (JEOL EX-500 spectroscopy, Tokyo, Japan), in conjunction with a comparison to published data.

#### Acid hydrolysis of isolated flavonoid glycosides

The method described by **Harborne et al.**^30^, was utilized to perform a comprehensive hydrolysis of the isolated flavonoid glycosides (compounds 2–5, 7, and 8). In this particular procedure, each isolated compound was separately dissolved in a solution of 5 mL dilute HCl in 80% methanol and subsequently heated at a temperature of 100ºC for a duration of two hours. To separate the aglycone, ethyl acetate was introduced into the reaction mixture, while the remaining aqueous phase contained the sugar. The identification of the sugar component was accomplished through the utilization of paper chromatography on Whatman No.1 paper sheets, alongside standard sugars. For the development of the chromatogram, a solvent system consisting of n-butanol, acetic acid, and water in a ratio of 4:1:5 was employed. The descending technique was adopted for this purpose. Following the application of aniline-phthalate spray and subsequent heating at a temperature of 110^ο^ C for a duration of 5 min, the sugar bands were successfully detected.

### Antimicrobial evaluation

All strains were sourced from the Microbial Genetics Laboratory at the National Research Centre in Egypt. The antimicrobial activity of the samples was assessed using the agar diffusion method against both gram-positive bacteria (*S. aureus (full name)* ATCC25923 and *Enterococcus faecalis* ATCC29212) and gram-negative bacteria (*Klebsiella pneumoniae* ATCC25175, *E. coli*(full name) ATCC25915, and *P. aeruginosa* (full name)ATCC10145). A nutrient agar medium was prepared, with the pH adjusted to 7.0 before sterilization. Following this, the nutrient agar was placed onto Muller Hinton agar plates, and once solidified, a bacterial lawn was established. Samples of 2 mg of each extracts were introduced and incubated at 37 °C for a duration of 24 h. The zones of inhibition for bacterial growth were subsequently measured^31^.

### Computational methods

#### Molecular Docking of synthesized compounds

All protein receptors were obtained from the RCSB Table 1. Following this, the structures of the target proteins underwent preprocessing utilizing PyMOL software. The structure of the compound was created using BIOVIA Draw. Open Babel^32^ was then employed to convert each compound into the mol2 format. Subsequently, AutoDock tools were utilized to transform the molecules into the pdbqt format. Prior to docking, ligand-centered maps were generated using AutoDock Vina^33^. The Discovery Studio program was used to analyze the two-dimensional interactions between the target proteins and the ligands. The ADMET properties of the compounds were calculated using BIOVIA Discovery Studio software^34^.

**Table 1:** Targets of antimicrobial proteins, PDB ids, active site coordinates, and reference.

Microorganism	Protein Targets	PDB ID	Resolutions	Active sit coordinates:			Reference
				X	Y	Z	
*S. aureus*	Dihydropteroate Synthetase	**1M17**	2.60 Å	19.96	5.08	20.12	Ciprofloxacin
*K. pneumoniae*	KPC-2 carbapenemase	**3ERT**	1.90 Å	27.52	63.35	−2.29	Ciprofloxacin
*P. aeruginosa*	LasR ligand-binding domain	**2UV0**	1.80 Å	72.07	36.33	11.54	Ciprofloxacin
*C. albicans*	Sterol 14-alpha demethylase	**5TZ1**	2.00 Å	19.96	5.08	20.12	Ciprofloxacin
*E. faecalis*	Penicillin-Binding Protein	**6MKI**	2.98 Å	27.52	63.35	−2.29	Ciprofloxacin
*E. coli*	GyrB24	**7P2M**	1.16 Å	72.07	36.33	11.54	Ciprofloxacin

#### Molecular dynamics (MD) simulation

Molecular dynamics (MD) simulation is extensively utilized to elucidate the binding interactions and affinities of protein-ligand complexes. In this investigation, MD simulations were conducted using GROMACS 2018 software to further validate the rationality and reliability of the docking outcomes. The protein’s topology was constructed employing the CHARMM36 force field parameters, while the topology of the compounds was generated via the Geoff server. Position restrictions were applied to the ligands. NVT and NPT equilibrations were executed for 1000 ps at 300 K under a pressure of 1.0 bar. Following the MD simulations, the Root Mean Square Deviation (RMSD), Root Mean Square Fluctuation (RMSF), and radius of gyration (Rg) were computed^35^. For plotting RMSD graphs, often utilize Grace/Xmgrace, a specialized 2D plotting tool compatible with Gromacs’.xvg output files, enabling direct visualization of time-dependent properties.

#### Statistical analysis

All experimental studies were conducted in replicates for statistical validity. Data were expressed as mean ± standard error. Results were considered statistically significant if the P-values were less than 0.05. All statistical analyses were performed using SPSS 17.0 software.

## Results

### Phytochemical investigation

#### Flavonoid screening

The methanol extract of *P. anisum* and *C. sativum* were treated individually with concentrated hydrochloric acid and magnesium, resulting in the development of red and orange colors, respectively. Furthermore, both fractions exhibited a yellow hue in the ammonia layer and formed a yellow precipitate upon the addition of 1% aluminum chloride. The presence of flavonoids in the methanol extract of both anise and coriander was further validated by the appearance of yellow coloration when sprayed with AlCl_3_ on TLC plates.

#### Flavonoids quantification

The quantitative assessment of total phenolic and flavonoid content in the methanol extracts of the aerial parts of *P. anisum* and *C. sativum* indicated that *P. anisum* possessed significantly higher levels of phenolics (53.1 ± 0.18 mg/g) and flavonoids (48.7 ± 0.21 mg/g) compared to *C. sativum*, which showed values of 43.5 ± 0.23 mg/g and 39.8 ± 0.19 mg/g, respectively (Table 2). These findings align with the earlier research conducted by **Bettaieb Rebey et al.**^36^ **and Tibebe et al.**^37^.

**Table 2:** Total phenolics and flavonoids in *P. anisum* and *C. sativum* methanol extracts.

Measured parameters	*P*. anisum	C. sativum
Total phenolics **(**mg/gm**)**	53.1 ± 0.18	43.5 ± 0.23
Total flavonoids **(**mg/gm**)**	48.7 ± 0.21	39.8 ± 0.19

Data were calculated from three replicates and expressed as mean ± S.D.

#### Flavonoids identification

**Fig. 1:**
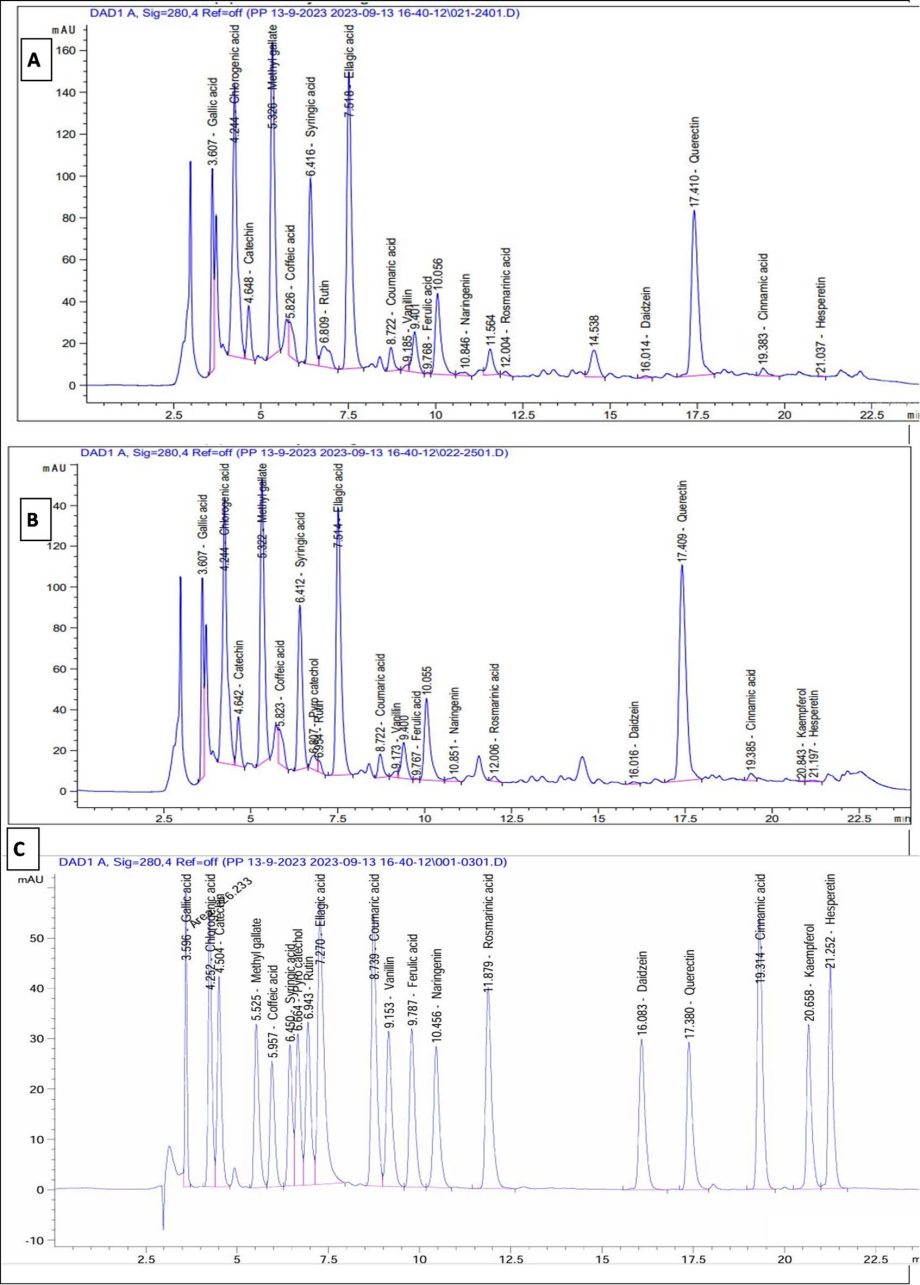
HPLC chromatogram of the methanol extract of *P. anisum* (A) and *C. sativum* (B), against standard phenolics and flavonoids (C).

The identification of phenolic acids and flavonoids in the methanol extracts of *P. anisum* and *C. sativum* was accompanied through HPLC analysis, comparing them to standard phenolics and flavonoids as depicted in Figure (1, A) for *P. anisum* and Figure (1, B) for *C. sativum*, and Figure (1, C) for standard phenolics and flavonoids. It is noteworthy to mention that querectin emerged as the predominant flavonoid in both *P. anisum* and *C. sativum*, with concentrations of 6970.39 µg/g and 9100.07 µg/g, respectively. Additionally, the primary phenolic acids detected in both plants were chlorogenic acid and ellagic acid with values of 8397.63 µg/g and 6203.23 µg/g for *P. anisum*, and 8311.54 µg/g and 5697.27 µg/g for *C. sativum* as presented in Table 3.

**Table 3:** Phenolic acids and flavonoids identification in the methanol extract of *P. anisum* and *C. sativum* from HPLC analysis.

NO	Standard compounds	*P*. anisum		C. sativum	
		Rt. (min.)	Conc. (µg/g)	Rt. (min.)	Conc. (µg/g)
1	Gallic acid	3.607	2125.54	3.607	2130.52
2	Chlorogenic acid	4.244	**8397.63**	4.244	**8311.54**
3	Catechin	4.648	2200.27	4.642	2071.90
4	Methyl gallate	5.326	3276.32	5.322	3015.91
5	Coffeic acid	5.826	691.26	5.823	694.89
6	Syringic acid	6.416	3116.25	6.412	2738.55
7	Pyro catechol	---	0.00	6.807	589.79
8	Rutin	6.809	1509.10	6.954	317.74
9	Ellagic acid	7.518	**6203.23**	7.514	**5697.27**
10	Coumaric acid	8.722	191.25	8.722	196.14
11	Vanillin	9.185	50.06	9.173	55.68
12	Ferulic acid	9.768	13.37	9.767	14.71
13	Naringenin	10.846	120.62	10.851	151.06
14	Rosmarinic acid	12.004	93.17	12.005	133.73
15	Daidzein	16.014	40.06	16.016	41.20
16	Querectin	17.410	**6970.39**	17.409	**9100.07**
17	Cinnamic acid	19.383	40.72	19.385	38.20
18	Kaempferol	---	0.00	20.843	3.57
19	Hesperetin	21.037	5.05	21.197	23.17

#### Structure Elucidation of isolated compounds

In view of the strong therapeutic benefits associated with *P. anisum* and *C. sativum*, a comprehensive study was conducted on methanol extracts from their aerial parts, which resulted in the isolation of eight flavonoids.

**Compound 1** was isolated as yellow powder and found to have a melting point of 312 ͦ C, ESI-MS revealed a molecular ion at *m/z* 316, which corresponds to the calculated molecular formula C_16_H_12_O_7_. Additionally, several notable fragments were observed at *m/z* 301, 287, 245, 153,142, 128, 108. Further analysis using ^1^H-NMR (500 MHz, CH_3_OH) δ: 6.22 (1H, d, J = 2.4 Hz, H-6), 6.37 (1H, d, J = 2.4 Hz, H-8), 7.69 (1H, d, J = 1.6 Hz, H-2`), 6.89 (1H, d, J = 7.9 Hz, H-5`), 7.68 (1H, dd, J = 7.9, 1.6 Hz, H-6`), 3.89 (3 H, s, OCH3). ^13^C-NMR (125 MHz, CH_3_OH, δ ppm): 156.8 (C2), 136.7 (C3), 175.8 (C4), 158.9 (C5), 98.7 (C6), 164.5 (C7), 93.1 (C8), 159.2 (C9), 102.4 (C10), 123.7 (C-1`),112.6 (C-2`), 146.2 (C-3`), 148.5 (C-4`), 115.8 (C-5`), 125.4 (C-6`), 57.4 (OCH3). The previous data were in agreement with that reported in the literature of **Cao et al.**^38^**.** Upon conducting a thorough analysis of the spectroscopic data and cross-referencing it with the existing literature, it has been ascertained that the isolated compound is isorhamnetin (3-methylquercetin).

**Compound 2** was obtained in the form of a yellow amorphous powder. Its melting point was determined to be 275 ͦ C. The ESI-MS analysis revealed a molecular weight of *m/z* 432, corresponding to the molecular formula C_21_H_20_O_10_. Additionally, several significant fragments were observed at m/z 287, 153, 147, 129. ^1^H-NMR data recorded at 500 MHz in CH_3_OH, displayed peaks at δ 6.23 (1H, d, J = 2.6 Hz, H-6), 6.31 (1H, d, J = 2.6 Hz, H-8), 7.94 (2 H, dd, J = 7.9, 2.2 Hz, H-2ʹ & H-6ʹ), 7.24 (2 H, dd, J = 7.9, 2.2 Hz, H-3ʹ & H-5ʹ), 5.13 (1H, d, J = 1.4 Hz, H-1″), 1.04 (3 H, d, J = 5.8 Hz, H-6″), and a range of signals from 4.12 to 3.15 ppm, corresponding to the sugar protons. The ^13^C-NMR data recorded at 125 MHz in CH_3_OH, displayed peaks at δ 156.7 (C-2), 135.2 (C-3), 181.5 (C-4), 164.1 (C-5), 97.6 (C-6), 167.4 (C-7), 94.2 (C-8), 159.6 (C-9), 105.2 (C-10), 119.7 (C-1ʹ), 128.7 (C-2ʹ & C-6ʹ), 114.7 (C-3ʹ & C-5ʹ), 158.2 (C-4ʹ), 102.5 (C-1″), 72.3 (C-2″), 71.2 (C-3″), 73.5 (C-4″), 69.8 (C-5″), 18.2 (Me-6″) **[39].** The glycosidic hydrolysis of the isolated compound proven the existence of rhamnose in its structure. The structure elucidation of the isolated compound was established as kaempferol 3-*O*-rhamnoside based on the spectral data, which are consistent with previous findings by **Rodríguez et al.**^40^.

**Compound** 3 was obtained in the form of a yellow amorphous powder. Its melting point was determined to be 270 ͦ C. The ESI-MS analysis showed a molecular weight of *m/z* 464, calculated for the molecular formula C_21_H_20_O_12_, with several significant fragments observed at *m/z* 318, 300, 287,245; ^1^H-NMR data recorded at 500 MHz in CH_3_OH, displayed peaks at δ 6.31(1H, d, 2.3 Hz, H-6), 6.45 (1H, d, 2.3 Hz, H-8), 7.13 (2 H, s, H-2’, 6’), 5.32 (1H, d, 1.6 Hz, H-1’’), 1.13 (3 H, d, J = 4.9 Hz, H-6″), 4.27–2.85 corresponding to the sugar protons. The ^13^C-NMR data recorded at 125 MHz in CH_3_OH, displayed peaks at δ 159.7 (C-2), 136.7 (C-3), 179.7 (C-4), 163.8 (C-5), 99.3 (C-6), 164.2 (C-7), 96.1 (C-8), 158.3 (C-9), 105.2 (C-10), 121.7 (C-1’), 111.0 (C-2’, 6’), 146.3 (C-3’, 5’), 139.2 (C-4’), 103.4 (C-1’’), 73.1 (C-2’’), 70.9 (C-3’’), 76.1 (C-4’’), 68.9 (C-5’’), 19.0 (C-6’’). The presence of rhamnose within the structure was confirmed through the glycosidic hydrolysis of the isolated compound. The MS, ^1^H-NMR, and ^13^C-NMR data were consistent with those of previous research^41^, confirming that the isolated compound was myricetin 3-*O*-rhamnoside, known as a myricitrin.

**Compound 4** was isolated as yellow powder; melting point 267 ͦ C. The ESI-MS analysis showed a molecular weight of *m/z* 448, calculated for the molecular formula C_21_H_20_O_11_, with several significant fragments observed at *m/z* 360, 286, 256. ^1^H-NMR (500 MHz, CH_3_OH) δ: 6.39 (1H, s, H-3), 6.81 (1H, d, J = 2.6 Hz, H-6), 6.63 (1H, d, J = 2.6 Hz, H-8), 7.39 (1H, d, J = 2.3 Hz, H-2′), 6.75 (1H, d, J = 7.9 Hz, H-5′), 7.52 (1H, dd, J = 7.9, 2.3 Hz, H-6′), 5.13 (1H, d, J = 1.5 Hz, H-1″); ^13^C-NMR (125 MHz, CH_3_OH, δ ppm): 165.2 (C-2), 102.6 (C-3), 181.7 (C-4), 160.7 (C-5), 98.6 (C-6), 162.8 (C-7), 95.2 (C-8), 156.9 (C-9), 104.8 (C-10), 122.3 (C-1′), 114.2 (C-2′), 146.2 (C-3′), 149.8 (C-4′),117.2 (C-5′), 118.6 (C-6′),99.7 (C-1″),74.1 (C-2″), 77.0 (C-3″), 70.4 (C-4″),78.4 (C-5″), 61.3 (C-6″). The confirmation of glucoside’s presence within the structure was achieved by conducting glycosidic hydrolysis on the isolated compound. By referring to the spectroscopic information documented in scientific literature^42^, the compound was successfully identified as luteolin 7-*O*-β-D-glucopyranoside.

**Compound 5** was obtained as yellow powder with melting point 176 ͦ C, EI-MS revealed molecular weight at *m/z* 610 for molecular formula C_21_H_20_O_11_, accompanied by other remarkable fragments at m/z 465, 303, 147, 129, 85. ^1^H-NMR (500 MHz, CH_3_OH) δ: 6.18 (1H, d, J = 2.1 Hz, H-6), 6.40 (1H, d, J = 2.1 Hz, H-8), 7.67 (1H, d, J = 1.4 Hz, H-2`), 6.88 (1H, d, J = 8.3 Hz, H-5`),7.60 (1H, dd, J = 8.3, 1.4 Hz, H-6`), 5.11 (1H, d, J = 1.9 Hz, H glucose), 4.48 (1H, d, J = 2.1 Hz, H rhamnose), 3.32–3.87 (sugar H). ^13^C-NMR (125 MHz, CH_3_OH, δ ppm): 157.3 (C-2),134.6 (C-3), 176.8 (C-4), 160.9 (C-5), 99.2 (C-6), 163.9 (C-7), 94.1 (C-8),155.8 (C-9), 104.2 (C-10), 123.2 (C-1′), 114.7 (C-2′), 145.3 (C-3′), 147.1 (C-4′),115.9 (C-5′), 120.8 (C-6′), 100.9 (C-1″),74.8 (C-2″), 75.7 (C-3″), 70.2 (C-4″), 76.2 (C-5″), 67.0 (C-6″), 99.8 (C-1′″), 69.6 (C-2′″), 68.3 (C-3′″), 72.3 (C-4′″), 67.8 (C-5′″), 18.4 (C-6′″). By subjecting the isolated compound to glycosidic hydrolysis, it was possible to verify the existence of glucose and rhamnose within its structure. The compound was recognized as rutin (quercetin 3-*O*-rutinoside) based on the spectroscopic information documented in the literature^43^^,^^44^.

**Compound 6** was isolated as yellow needles, with melting point 315 ͦ C, ESI-MS exhibited molecular weight at *m/z* 302 for molecular formula C_15_H_10_O_7_, along with other noteworthy fragments at *m/z* 257, 229, 201, 153, 127. ^1^H-NMR (500 MHz, CH_3_OH) δ: 6.23(1H, d, J = 2.6 Hz, H-6), 6.35(1H, d, J = 2.6 Hz, H-8), 7.53(1H, d, J = 2.7 Hz, H-2’), 6.54(1H, d, J = 7.4 Hz, H-5’), 7.63(1H, dd, J = 7.4, 2.7 Hz, H-6’). ^13^C-NMR (125 MHz, CH_3_OH, δ ppm): 156.7 (C-2), 134.6 (C-3), 180.1 (C-4), 163.6 (C-5), 99.4 (C-6), 166.4 (C-7), 94.3 (C-8), 159.2 (C-9), 104.9 (C-10), 121.4 (C-1`), 115.8 (C-2`), 145.3 (C-3`), 149.6 (C-4`), 116.8 (C-5`), 121.7 (C-6`). The spectral findings concurred with the data recorded in previous research on quercetin^45^.

**Compound 7** was isolated as yellowish white powder with melting point 180 ͦ C, ESI-MS exhibited molecular weight at *m/z* 448 (100%) for molecular formula C_21_H_20_O_11_, along with other noteworthy fragments at m/z 302, 271, 255, 243, 227, 107. ^1^H-NMR (500 MHz, CH_3_OH) δ: 6.18 (1H, d, J = 2.3 Hz, H-6), 6.29 (1H, d, J = 2.3 Hz, H-8), 7.49 (1H, d, J = 1.2 Hz, H-2`), 6.63 (1H, d, J = 7.8 Hz, H-5`),7.58 (1H, dd, J = 7.8, 1.2 Hz, H-6`), 5.09 (1H, d, J = 2.1 Hz, H-1``), 1.02 (3 H, d, J = 5.7 Hz, H-6``), 4.19–3.17 (sugar H). ^13^C-NMR (125 MHz, CH_3_OH, δ ppm): 157.3 (C-2), 135.2 (C-3), 179.0 (C-4), 162.4 (C-5), 98.7 (C-6), 165.2 (C-7), 93.7 (C-8), 158.7 (C-9), 105.6 (C-10), 120.8 (C-1`), 116.2 (C-2`), 146.5 (C-3`), 150.1 (C-4`), 117.3 (C-5`), 122.3 (C-6`), 103.4 (C-1``), 68.5 (C-2``), 69.8 (C-3``), 70.2 (C-4``), 70.9 (C-5``), 19.4 (C-6``). Verification of the presence of rhamnose in the structure of the isolated compound was achieved by subjecting it to glycosidic hydrolysis. The spectral results were in line with the information documented in earlier researches on quercetrin (Quercetin 3-*O*-rhamnoside)^46^^,^^47^.

**Compound 8** was isolated as yellow crystals with a melting point of 188 ͦ C. The molecular weight of the compound was determined to be *m/z* 578 by ESI-MS, which is consistent with the molecular formula C_27_H_30_O_14_, in addition to other notable fragments at m/z 432, 287, 147, 129. ^1^H-NMR (500 MHz, CH_3_OH) displayed peaks at δ 6.39 (1H, d, J = 2.8 Hz, H-6), 6.67 (1H, d, J = 2.8 Hz, H-8), 7.84 (2 H, dd, J = 8.3, 2.5 Hz, H-2ʹ & H-6ʹ), 7.13 (2 H, dd, J = 8.3, 2.5 Hz, H-3ʹ & H-5ʹ), 5.24 (1H, d, J = 1.6 Hz, (3-O-Rha)−1``), 5.36 (1H, d, J = 1.7 Hz, (7-O-Rha)−1``), 1.04 (3 H, d, J = 5.7 Hz, (3-O-Rha)-CH3), 1.13 (3 H, d, J = 5.9 Hz, (7-O-Rha)-CH3), and a range of signals from 4.25 to 3.11 ppm corresponding to the sugar protons. ^13^C-NMR data was recorded at 125 MHz, in CH_3_OH solvent, and the chemical shifts (δ ppm) were observed as follows: 159.2 (C-2), 134.8 (C-3), 177.9 (C-4), 162.5 (C-5), 99.1 (C-6), 163.1 (C-7), 95.1 (C-8), 155.8 (C-9), 105.8 (C-10), 120.7 (C-1`), 129.8 (C-2`, 6`), 117.0 (C-3`, 5`), 159.6 (C-4`). Additionally, the 3-Rhamnose protons were observed at 104.2 (C-1``), 71.5 (C-2``), 70.3 (C-3``), 69.8 (C-4``), 68.7 (C-5``), 17.1 (Me-6``). Furthermore, the 7-Rhamnose protons were detected at 101.8 (C-1``), 71.0 (C-2``), 70.1 (C-3``), 72.4 (C-4``), 68.6 (C-5``), 16.8 (Me-6``). Through the process of glycosidic hydrolysis, the existence of rhamnose within the structure of the isolated compound was established. Upon comparison with the reported data, it can be concluded that the mass spectrometry (MS), ^1^H-NMR, and ^13^C-NMR results are consistent with the documented characteristics of Kaempferol 3,7-*O*-dirhamnoside as described by **Liang et al.**^48^.

This study provides the first documentation of these eight flavonoids that have been isolated from the aerial parts of anise and coriander.

### Antimicrobial activity

**Fig. 2:**
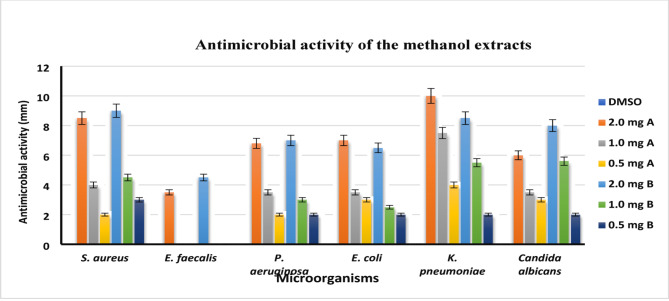
Percentage of antimicrobial activity of the methanol extracts of *P. anisum* and *C. sativum* aerial parts evaluated by well diffusion method against *S. aureus* ATCC25923, *E. faecalis* ATCC29212, *K. pneumoniae* ATCC25175, *P. aeruginosa* ATCC10145, *E. coli* ATCC25915 and *C. albicans.*

**Fig. 3:**
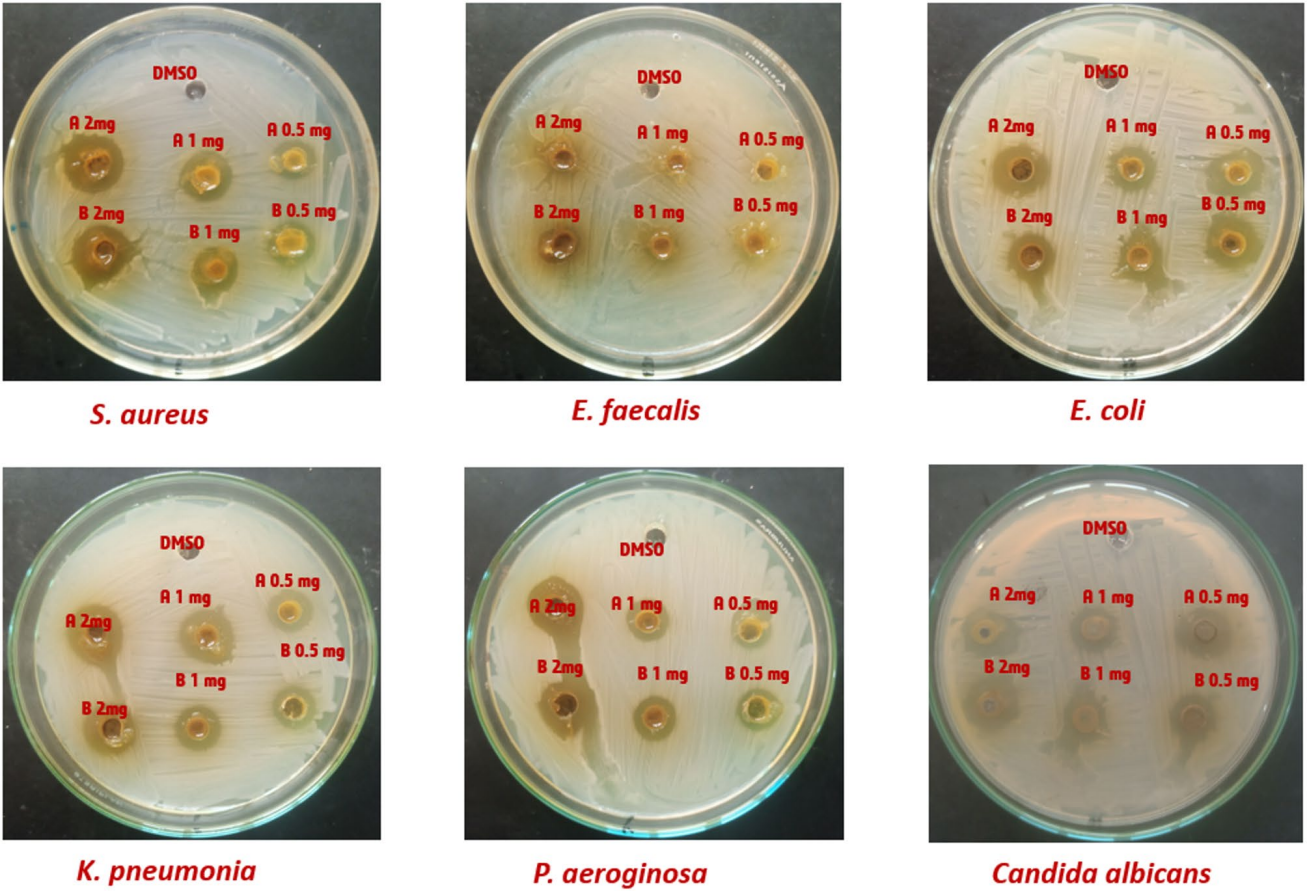
Antimicrobial activity of the methanol extracts of *P. anisum* and *C. sativum* aerial parts evaluated by well diffusion method against *S. aureus* ATCC25923, *E. faecalis* ATCC29212, *K. pneumoniae* ATCC25175, *P. aeruginosa* ATCC10145, *E. coli* ATCC25915 and *Candida albicans.*

The effectiveness of methanol extract of *P. anisum* and *C. sativum* aerial parts with different concentrations (2.0, 1.0, and 0.5 mg/mL) was tested against a range of microorganisms, including gram-negative bacteria (*E. coli*,* K. pneumoniae*, and *P. aeruginosa*), gram-positive bacteria (*S. aureus* and *E. faecalis*), and *C. albicans*. Our research discovered that extract **A (***P. anisum*), with concentrations of (2.0, 1.0, and 0.5 mg/mL), exhibited strong antimicrobial properties. It showed values of (7.00 ± 0.05, 3.50 ± 0.00, and 3.00 ± 0.01) against *E. coli*, (8.50 ± 0.09, 4.00 ± 0.10, and 2.00 ± 0.00) against *S. aureus*, (6.80 ± 0.01, 3.50 ± 0.00, and 2.00 ± 0.00) against *P. aeruginosa*, (10.0 ± 0.10, 7.50 ± 0.08, and 4.00 ± 0.00) against *K. pneumoniae*, (3.50 ± 0.08, 0.0, and 0.0) against *E. faecalis*, and (6.00 ± 0.00, 3.50 ± 0.00, and 3.00 ± 0.01) against *C. albicans*. Notably, the concentrations of 2.0 and 1.0 mg/mL showed significant inhibition against *K. pneumoniae* and *S. aureus*, in addition, it demonstrated a lower antimicrobial activity, registering 3.50 ± 0.08 against *E. faecalis* at a concentration of 2.0 mg/mL. as illustrated in Figures (2&3) and **Table ****S1**. Additionally, our results indicate that extract **B (***C. sativum*), with concentrations of (2.0, 1.0, and 0.5 mg/mL), demonstrated increased antibacterial effectiveness. It yielded values of (6.55 ± 0.08, 2.50 ± 0.00, and 2.00 ± 0.0) against *E. coli*, (9.00 ± 0.20, 4.50 ± 0.04, and 3.0 ± 0.10) against *S. aureus*, and (8.55 ± 0.0, 5.50 ± 0.10, and 2.00 ± 0.10) against *K. pneumoniae*. Furthermore, the extract exhibited a moderate antimicrobial impact, with values of (7.0 ± 0.10, 3.0 ± 0.0, and 2.00 ± 0.08) against *P. aeruginosa*, (8.0 ± 0.20, 5.60 ± 0.0, and 2.0 ± 0.06) against *C. albicans* at concentrations of 2.0, 1.0, and 0.5 mg/mL, respectively. Moreover, it showed lower antimicrobial activity, registering 4.50 ± 0.07 against *E. faecalis* at a concentration of 2.0 mg/mL. Thus, our findings suggest that the methanol extracts containing phenolic compounds possess promising antimicrobial properties against the studied pathogens. Additionally, The MIC data presented in the **Table S2** reveals several critical observations and concerns. Notably, both extracts (A and B) exhibit identical MIC values across most tested microorganisms (0.25 mg/mL for *E. coli*, *S. aureus*, *P. aeruginosa*, *K. pneumoniae*, and *C. albicans*, and 2.0 mg/mL for *E. faecalis*). MIC values generally reflect variability in microbial susceptibility. The elevated MIC for *E. faecalis* (2.0 mg/mL) aligns with its weaker inhibition zones, suggesting reduced susceptibility, though the identical MIC for both extracts warrants exploration of shared inhibitory mechanisms.

### Computational analysis

#### Docking and molecular interaction of identified compounds

**Fig. 4:**
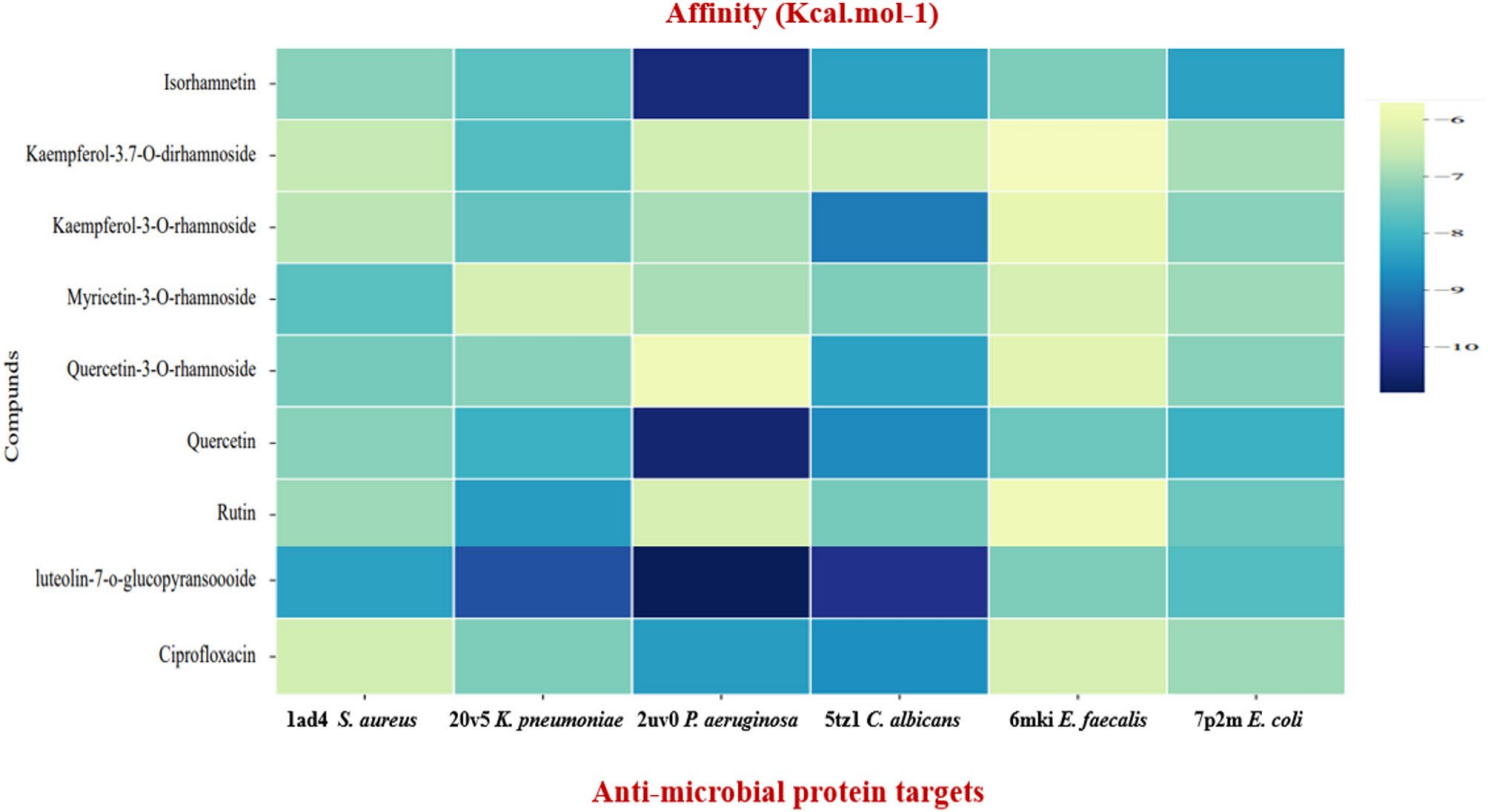
Heatmap of binding affinity of compounds with targets of antimicrobial proteins.

To explore the binding interactions between the identified compounds and protein targets linked to antibacterial activities, molecular docking analyses were conducted. This investigation sought to offer insights into the efficacy of the compounds. The outcomes of the docking experiments, as illustrated in Table 4 and Fig. 4, showcased the assessment of binding affinities between the compounds and three antimicrobial receptors.

**Table 4:** Binding affinity of ligands with targets of antimicrobial activity.

No	Ligands	Affinity (Kcal.mol-1)					
		S. aureus	K. pneumoniae	*P*. aeruginosa	C. albicans	E. faecalis	E. coli
		(PDB.ID: 1AD4)	(PDB.ID: 2OV5)	(PDB.ID: 2UV0)	(PDB.ID: 5TZ1)	(PDB.ID: 6MKI)	(PDB.ID: 7P2M)
1	**Isorhamnetin**	−7.20	−7.70	−10.40	−8.40	−7.30	−8.40
2	**Kaempferol 3**,**7-*O*-dirhamnoside**	−6.60	−7.80	−6.40	−6.40	−5.70	−6.90
3	**Kaempferol 3-** ***O*** **-rhamnoside**	−6.70	−7.60	−6.90	−9.00	−6.00	−7.20
4	**Myricetin 3-** ***O*** **-rhamnoside**	−7.70	−6.30	−6.90	−7.30	−6.30	−7.00
5	**Quercetin 3-** ***O*** **-rhamnoside**	−7.40	−7.20	−5.80	−8.40	−6.10	−7.20
6	**Quercetin**	−7.20	−8.10	−10.50	−8.80	−7.50	−8.10
7	**Rutin**	−7.00	−8.50	−6.30	−7.40	−5.80	−7.50
8	**Luteolin 7-** ***O*** **-glucopyransoide**	−8.40	−9.60	−10.80	−10.20	−7.30	−7.80
9	**Ciprofloxacin**	−6.40	−7.30	−8.50	-	−6.30	−7.00
10	**Fluconazole**	-	-	-	−7.20	-	-

#### Docking and interaction with dihydropteroate synthase of *S. aureus*

**Fig. 5:**
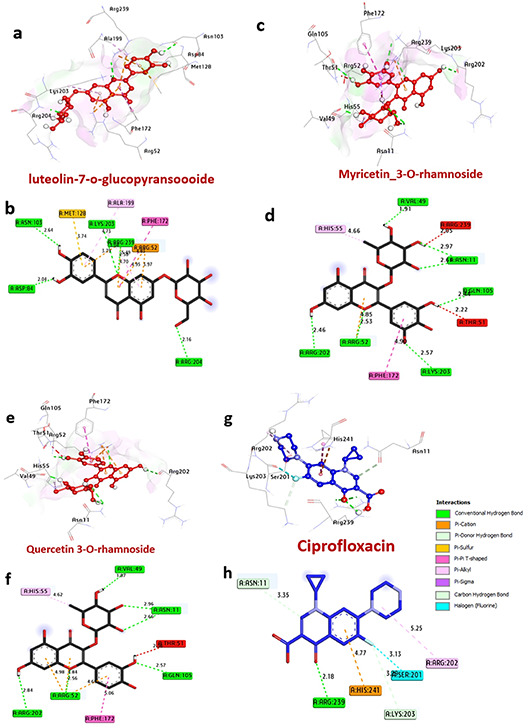
3D representations of the compound at the binding pocket of dihydropteroate synthase of *S. aureus* (PDB: ID 1AD4). (**a** and **b**) luteolin 7-*O*-glucopyransoide, (**c** and **d**) Myricetin 3-*O*-rhamnoside, (**e** and **f**) Quercetin 3-*O*-rhamnoside and (**g** and **h**) ciprofloxacin.

Dihydropteroate synthase plays a role in the folate synthesis pathway, and inhibition of this enzyme is needed for bacterial growth. The compounds luteolin7-*O*-glucopyranoside, myricetin 3-*O*-rhamnoside, and quercetin 3-*O*-rhamnoside exhibited favorable binding energies of −8.40, −7.70, and − 7.40 kcal/mol, respectively, compared to Ciprofloxacin (−6.40 kcal/mol) **(**Table 5**).** These compounds formed hydrogen bonds with multiple amino acids such as Arg204, Arg239, Asn103, Asp84, Val49, Asn11, Gln105, Arg52, and Arg202. Additionally, various hydrophobic interactions within the active pocket were observed, including alkyl bonds with Ala199, Met128, Lys203, His55, Pi-Pi interactions with Phe172, Pi-cation interactions with Arg52 and Arg239, Pi-Sulfur interactions with Met128, and Pi-sigma interactions with Arg204. Furthermore, amino acids Lys203, Phe172, and Val49 in the catalytic site were found to enhance the binding affinity. Overall, these compounds are likely to exert their antibacterial activity by inhibiting the dihydropteroate synthase enzyme in *S. aureus*. The three-dimensional structures of the compounds situated at the binding pocket of dihydropteroate synthase in *Staphylococcus aureus* (PDB: ID 1AD4) are illustrated in Fig. 5. Panels (a and b) correspond to luteolin 7-*O*-glucopyranoside, (c and d) to myricetin 3-*O*-rhamnoside, (e and f) to quercetin 3-*O*-rhamnoside, and (g and h) to ciprofloxacin.

**Table 5:** Molecular interactions with amino acids of dihydropteroate synthase of *S. aureus* (PDB: ID 1AD4).

	Protein	Ligand	3D Structure	Hydrophilic Interactions	Hydrophobic Contacts			No. of H-Bonds	No. of Total Bonds	affinity kcal mol-1
				Residue (H- Bond)	Length	Residue (Bond type)	Length			
1	**Dihydropteroate synthase of** ***S. aureus***	**Luteolin7-** ***O*** **-glucopyransoide**		Arg204 (H- Bond) Lys203 (H- Bond) Arg239 (H- Bond) Asn103 (H- Bond) Asp84 (H- Bond)	2.16 4.73 4.70 2.64 2.04	Phe172, (Pi-Pi shaped) Arg52, (Pi-cation) Arg52, (Pi-cation) Ala199, (Pi-alkyl) Met128, (Pi-Sulfur) Ala199, (Pi-alkyl)	4.81 4.95 3.97 4.73 3.74 2.58	**5**	**11**	**−8.40**
2		**Myricetin 3-** ***O*** **-rhamnoside**		Val49 (H- Bond) Asn11 (H- Bond) Asn11 (H- Bond) Gln105 (H- Bond) Lys203 (H- Bond) Arg52 (H- Bond) Arg202 (H- Bond)	1.91 2.97 2.60 2.44 2.57 2.53 2.46	His55, (Pi-alkyl) Arg52, (Pi- cation) Phe172, (Pi-Pi shaped)	4.66 4.85 4.90	7	**10**	**−7.70**
3		**Quercetin 3-** ***O*** **-rhamnoside**		Val49 (H- Bond) Asn11 (H- Bond) Asn11 (H- Bond) Gln105 (H- Bond) Arg52 (H- Bond) Arg202 (H- Bond)	1.87 2.96 2.66 2.57 2.56 2.84	His55, (Pi-alkyl) Arg52, (Pi-cation) Arg52, (Pi- cation) Phe172, (Pi-Pi shaped)	4.62 4.66 4.98 5.06	**6**	**10**	**−7.40**
4		**Ciprofloxacin**		Arg239 (H- Bond)	2.18	Ser201, (Halogen) Arg202, (Pi-alkyl) His241, (Pi-cation) Lys203, (CH-bond) Asn11, (CH-bond)	3.13 5.25 4.77 3.55 3.35	**1**	**6**	**−6.10**

#### Docking and molecular interaction studies of LasR protein in *P. aeruginosa*

**Fig. 6:**
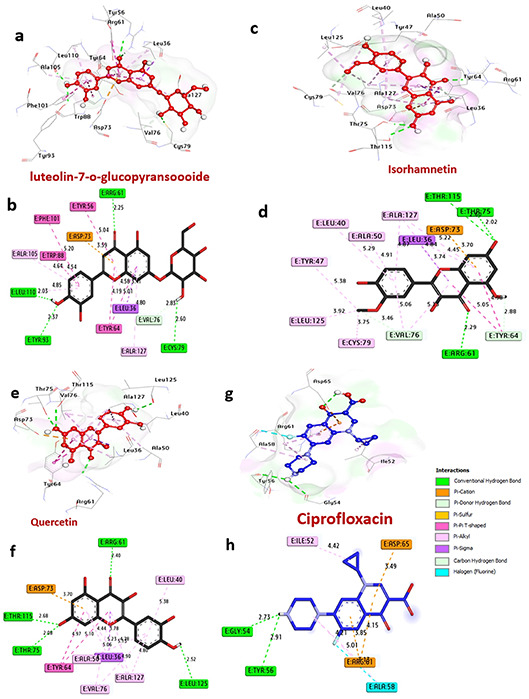
3D representations of compound at the binding pocket of LasR protein in *P. aeruginosa* (PDB: ID 2UV0): (**a** and **b**) luteolin 7-*O*-glucopyransoide, (**c** and **d**) Isorhamnetin, (**e** and **f**) Quercetin and (**g** and **h**) Ciprofloxacin.

The LasR protein functions as a transcriptional regulator for pathogenicity in *P. aeruginosa*. The docking results of compounds and ciprofloxacin are detailed in Table 6 and Fig. 6. Notably, among the compounds, luteolin7-*O*-glucopyransoide, isorhamnetin, and quercetin exhibited the highest affinity interactions, −10.80, −7.90, and − 10.50 kcal/mol, respectively, surpassing ciprofloxacin’s affinity of −8.50 kcal/mol. These compounds were found to form hydrogen bonds with key amino acids such as Cys79, Arg61, Tyr93, Thr115, Thr75, Leu125, and Thr75. Additionally, several hydrophobic interactions occurred within the activity pocket, including alkyl bonds with Ala127, Ala105, Asp65, Leu17, Cys79, Leu125, Tyr47, Ala50, Leu40, Val76, Pi-cation bonds with Asp73, Pi-sigma bonds with Leu36, Pi-Pi T shaped bonds with Tyr56, Phe101, Trp88, Tyr64, and Carbon H bonds with Gln24, Tyr64, and Val76. Furthermore, Thr75, Thr115, and Arg61 residues at the catalytic site were observed to enhance the binding affinity.

**Table 6:** Molecular interactions of ligands with LasR protein in *P. aeruginosa*.

	Protein	Ligand	3D Structure	Hydrophilic Interactions	Hydrophobic Contacts			No. of H-Bonds	No. of Total Bonds	affinity kcal mol-1
				Residue (H- Bond)	Length	Residue (Bond type)	Length			
1	**LasR protein in** ***P. aeruginosa*** (PDB: ID 2UV0)	Luteolin 7-*O*-glucopyransoide		Cys79, (H- Bond) Arg61, (H- Bond) Tyr93, (H- Bond) Leu110, (H- Bond)	2.60 2.25 2.37 2.03	Leu36, (Pi-sigma) Asp73, (Pi-cation) Tyr56, (Pi-Pi T shaped) Phe101, (Pi-Pi T shaped) Trp88, (Pi-Pi T shaped) Tyr64, (Pi-Pi T shaped) Ala127, (Pi-alkyl) Ala105, (Pi-alkyl)	5.01 3.59 5.04 5.20 5.20 4.64 4.19 4.80 4.64	**4**	12	**−10.80**
2		Isorhamnetin		Arg61, (H- Bond) Thr115, (H- Bond) Thr75, (H- Bond)	2.29 2.60 2.02	Tyr64, (Pi-Pi T shaped) Tyr64, (Pi-Pi T shaped) Cys79, (Pi-alkyl) Leu125, (Pi-alkyl) Tyr47, (Pi-alkyl) Ala50, (Pi-alkyl) Leu40, (Pi-alkyl) Ala127, (Pi-alkyl) Val76, (Pi-alkyl) Asp73, (Pi-cation) Leu36, (Pi-sigma) Tyr64, (Carbon H bond) Val76, (Carbon H bond)	2.88 5.05 3.75 3.92 5.38 5.29 4.91 4.87 5.06 3.70 3.74 2.88 3.46	**3**	16	**−7.90**
3		Quercetin		Leu125, (H- Bond) Thr115, (H- Bond) Thr75, (H- Bond)	2.52 2.68 2.08	Tyr64, (Pi-Pi T shaped) Tyr64, (Pi-Pi T shaped) Ala50, (Pi-alkyl) Leu40, (Pi-alkyl) Ala127, (Pi-alkyl) Val76, (Pi-alkyl) Asp73, (Pi-cation) Leu36, (Pi-sigma)	5.10 4.97 5.13 5.38 4.80 4.90 3.70 3.78	**3**	12	**−10.50**
4		Ciprofloxacin		Gly54, (H- Bond) Tyr56, (H- Bond)	2.73 2.91	Ile52, (Pi-alkyl) Asp65, (Pi-cation) Arg61, (Pi-cation) Arg61, (Pi-cation) Arg61, (Pi-alkyl) Ala58, (Pi-alkyl) Ala58, (Halogen)	4.42 3.49 4.15 3.85 4.21 5.01 3.13	**2**	9	**−8.50**

#### Docking and molecular interaction studies with DNA gyrase of *E.coli*

**Fig. 7:**
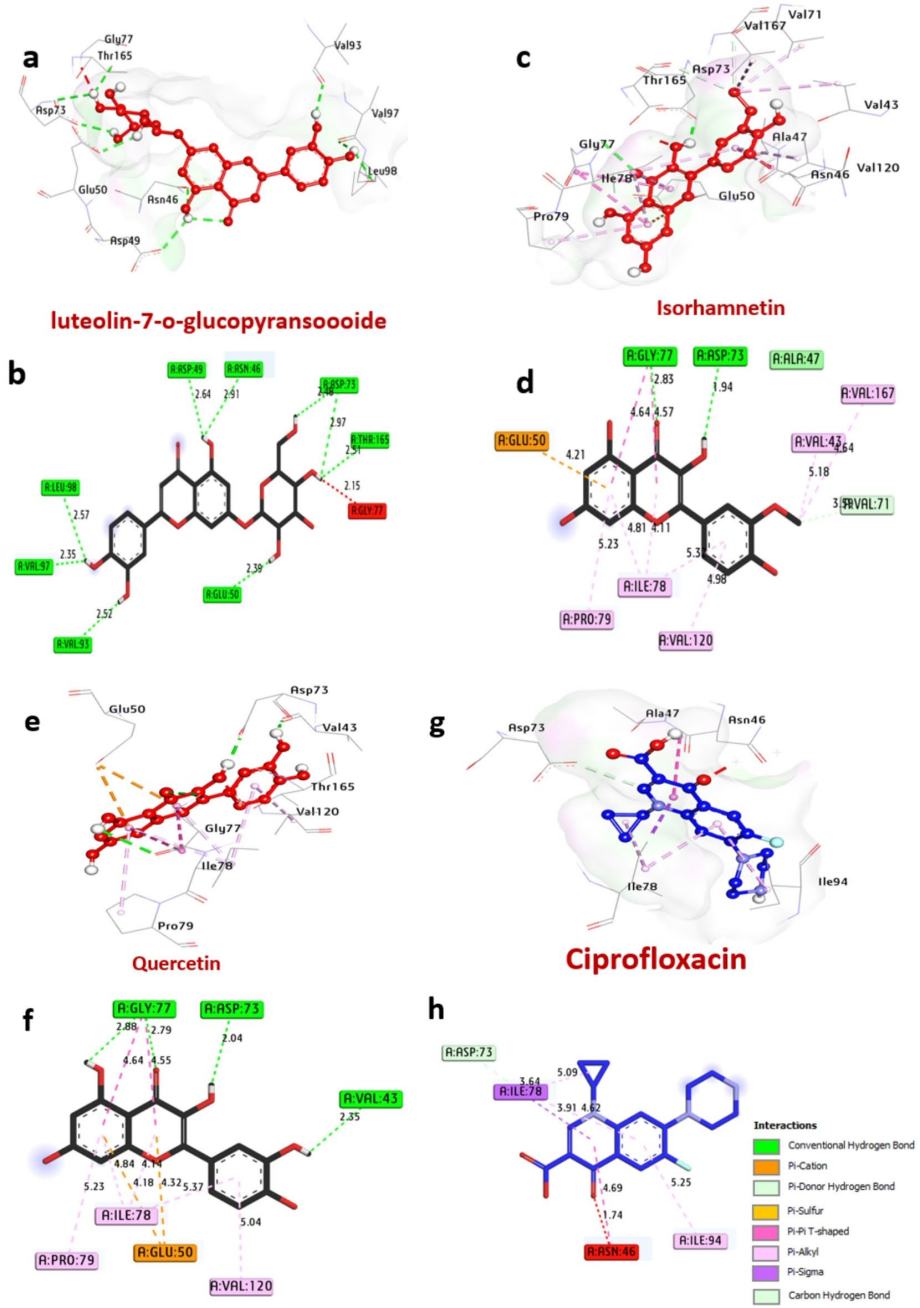
3D representations of compounds at the binding pocket of DNA Gyrase of *E.coli* (PDB: ID 7P2M): (**a** and **b**) luteolin-7-*O*-glucopyransoide, (**c** and **d**) Isorhamnetin, (**e** and **f**) Quercetin and (**g** and **h**) Ciprofloxacin.

DNA gyrases are an enzyme found in bacteria, including *E. coli*, that plays a crucial role in DNA replication and transcription. According to the analysis of docking results (Table 7; Fig. 7), among all compounds, the best bacteria inhibitors luteolin-7-*O*-glucopyransoide, isorhamnetin and quercetin have the greatest affinity interaction, −7.80, −8.10 and − 8.10 kcal/mol, respectively compared with control ciprofloxacin − 7.00 kcal/mol). Compounds formed hydrogen bonds with the key amino acids: Asp73, Thr165, Glu50, Asn46, Asp49, Leu98, Val97, Val93, Ile94, Val118, Asp73, Gly77, Val43, Gly77. Also, several hydrophobic bond interactions within the activity pocket were formed including alkyl bonds with Val43, Val120, Val167, Ile78, Val167, and Pro79, (Pi-Pi T shaped) with Gly77 and Ile94, (Carbon-H bond) with Leu98 and Val71, (pi-Sigma) with Asn46, (pi-cation) with Glu50. Furthermore, it can be observed that the amino acids Asp73, Gly77, and Val43, in the catalytic site enhance the binding affinity.

**Table 7:** Molecular interactions of ligands with amino acids of DNA gyrase of *E.coli* (PDB: ID 7P2M).

NO	Protein	Ligand	3D Structure	Hydrophilic Interactions		Hydrophobic Contacts		No. of H-Bonds	No. of Total Bonds	affinity kcal mol-1
				Residue (H- Bond)	Length	Residue (Bond type)	Length			
1	**DNA Gyrase of** ***E.coli*** **(PDB: ID 7P2M)**	Luteolin 7-*O*-glucopyransoide		Asp73, (H- Bond) Thr165, (H- Bond) Asp73, (H- Bond) Glu50, (H- Bond) Asn46, (H- Bond) Asp49, (H- Bond) Leu98, (H- Bond) Val97, (H- Bond) Val93, (H- Bond)	2.48 2.51 2.97 2.39 2.91 2.64 2.57 2.35 2.52	Gly77, (Unfavorable)	2.15	**9**	**10**	**−7.80**
2		Isorhamnetin		Asp73, (H- Bond) Gly77, (H- Bond)	1.94 2.83	Val167, (alkyl) Val43, (alkyl) Ile78, (alkyl Ile78, (alkyl) Val120, (alkyl) Pro79, (alkyl) Gly77, (Pi-Pi T shaped) Gly77, (Pi-Pi T shaped) Val71, (C-H bond) Glu50, (pi-cation)	4.64 5.18 5.30 4.11 4.98 5.23 4.64 4.57 3.20 4.21	**2**	**14**	**−8.10**
3		Quercetin		Val43, (H- Bond) Asp73, (H- Bond) Gly77, (H- Bond) Gly77, (H- Bond)	2.35 2.04 2.79 2.88	Ile78, (alkyl) Ile78, (alkyl Ile78, (alkyl) Val120, (alkyl) Pro79, (alkyl) Gly77, (Pi-Pi T shaped) Gly77, (Pi-Pi T shaped) Glu50, (pi-cation) Glu50, (pi-cation)	5.37 4.18 4.84 5.04 5.23 4.64 4.55 4.32 4.18	**4**	**13**	**−8.10**
4		Ciprofloxacin		**-**	-	Asn46, (Unfavorable) Ile94, (alkyl) Ile78, (alkyl) Ile78, (alkyl Asn46, (Pi-Pi T shaped) Ile78, (pi-Sigma) Asp73, (C-H bond)	1.81 5.20 4.61 3.87 4.73 3.87 3.70	**0**	**7**	**−7.00**

#### Docking and interaction of KPC-2 carbapenemase of *K. pneumoniae*

**Fig. 8:**
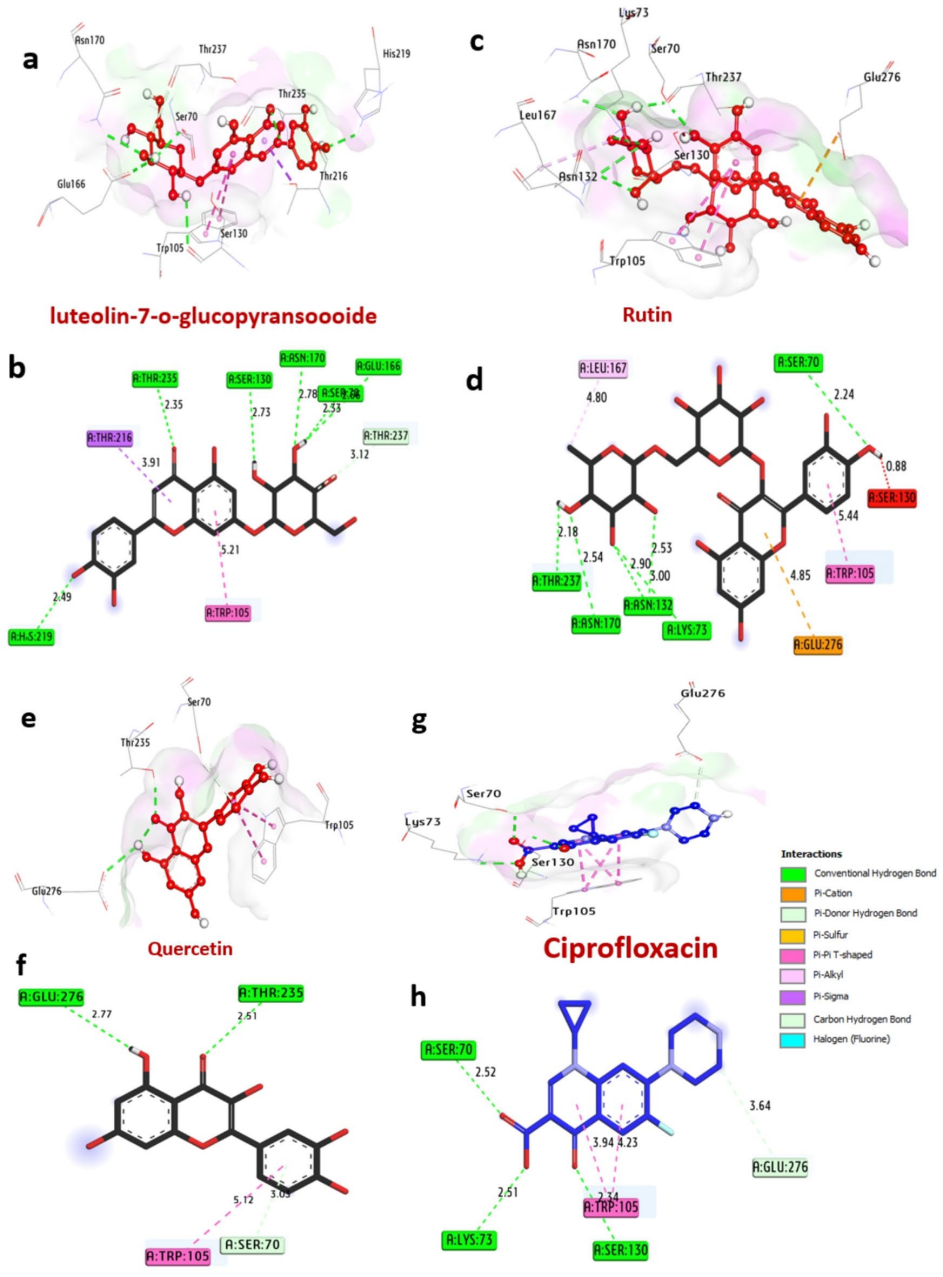
3D representations of compounds at the binding pocket of KPC-2 carbapenemase of *K. pneumoniae* (PDB: ID 2OV5): (**a** and **b**) luteolin-7-o-glucopyransoide, (**c** and **d**) Rutin, (**e** and **f**) Quercetin and (**g** and **h**) Ciprofloxacin.

KPC-2 carbapenemase is an important enzyme in *K. pneumoniae* which is an enzyme that confers resistance to carbapenem antibiotics, a class of antibiotics against multidrug-resistant bacteria. The docking findings with the selected molecules are shown in Table 8; Fig. 8. Among all compounds, the best bacteria inhibitors luteolin7-*O*-glucopyransoide, rutin and quercetin have the greatest affinity interaction, −9.60, −8.50, and − 8.10 kcal/mol, respectively compared with ciprofloxacin (−7.30 kcal/mol). Compounds formed hydrogen bonds with the key amino acids: His219, Thr235, Ser130, Asn170, Glu166, Thr215, Lys73, Asn132, Ser70, Thr237, Glu276. Also, several hydrophobic bond interactions within the activity pocket were formed including alkyl bonds with Leu167, (Pi-Pi T shaped) with Trp105, (Pi-sigma) with Thr216, (Carbon H-bond) with Thr237, (Pi-cation) with Glu276. Furthermore, it can be observed that the amino acids Ser130, Thr235, and Ser70, in the catalytic site enhance the binding affinity.

**Table 8:** Molecular interactions of ligands with amino acids of KPC-2 carbapenemase of *K. pneumoniae* (PDB: ID 2OV5).

No	Protein	Ligand	3D Structure	Hydrophilic Interactions		Hydrophobic Contacts		No. of H-Bonds	No. of Total Bonds	affinity kcal mol-1
				Residue (H- Bond)	Length	Residue (Bond type)	Length			
1	**KPC-2 carbapenemase of** ***K. pneumoniae*** **(PDB: ID 2OV5)**	luteolin7-*O*-glucopyransoide		**His219**,** (H- Bond)** **Thr235**,** (H- Bond)** **Ser130**,** (H- Bond)** **Asn170**,** (H- Bond)** **Glu166**,** (H- Bond)** **Ser130**,** (H- Bond)**	**2.49** **2.35** **2.73** **2.78** **2.33** **2.55**	**Trp105**,** (Pi-Pi T shaped)** **Thr216**,** (Pi-sigma)** **Thr237**,** (C-H bond)**	**5.21** **3.91** **3.12**	6	9	**−9.60**
2		Rutin		**Ser70**,** (H- Bond)** **Thr237**,** (H- Bond)** **Asn132**,** (H- Bond)** **Asn132**,** (H- Bond)** **Lys73**,** (H- Bond)** **Asn170**,** (H- Bond)**	**2.24** **2.18** **3.00** **2.90** **2.53** **2.54**	**Trp105**,** (Pi-Pi T shaped)** **Leu167**,** (alkyl)** **Glu276**,** (Pi-cation)**	**5.44** **4.80** **4.85**	6	10	**−8.50**
3		Quercetin		**Thr235**,** (H- Bond)** **Glu276**,** (H- Bond)**	**2.51** **2.77**	**Trp105**,** (Pi-Pi T shaped)** **Ser70**,** (C-H bond)**	**5.12** **3.03**	2	4	**−8.10**
4		Ciprofloxacin		**Ser130**,** (H- Bond)** **Lys73**,** (H- Bond)** **Ser70**,** (H- Bond)**	**2.34** **2.51** **2.52**	**Trp105**,** (Pi-Pi T shaped)** **Trp105**,** (Pi-Pi T shaped)**	**4.23** **3.94**	3	5	**−7.30**

#### Docking and interaction with Penicillin-Binding protein of *E. faecalis*

**Fig. 9:**
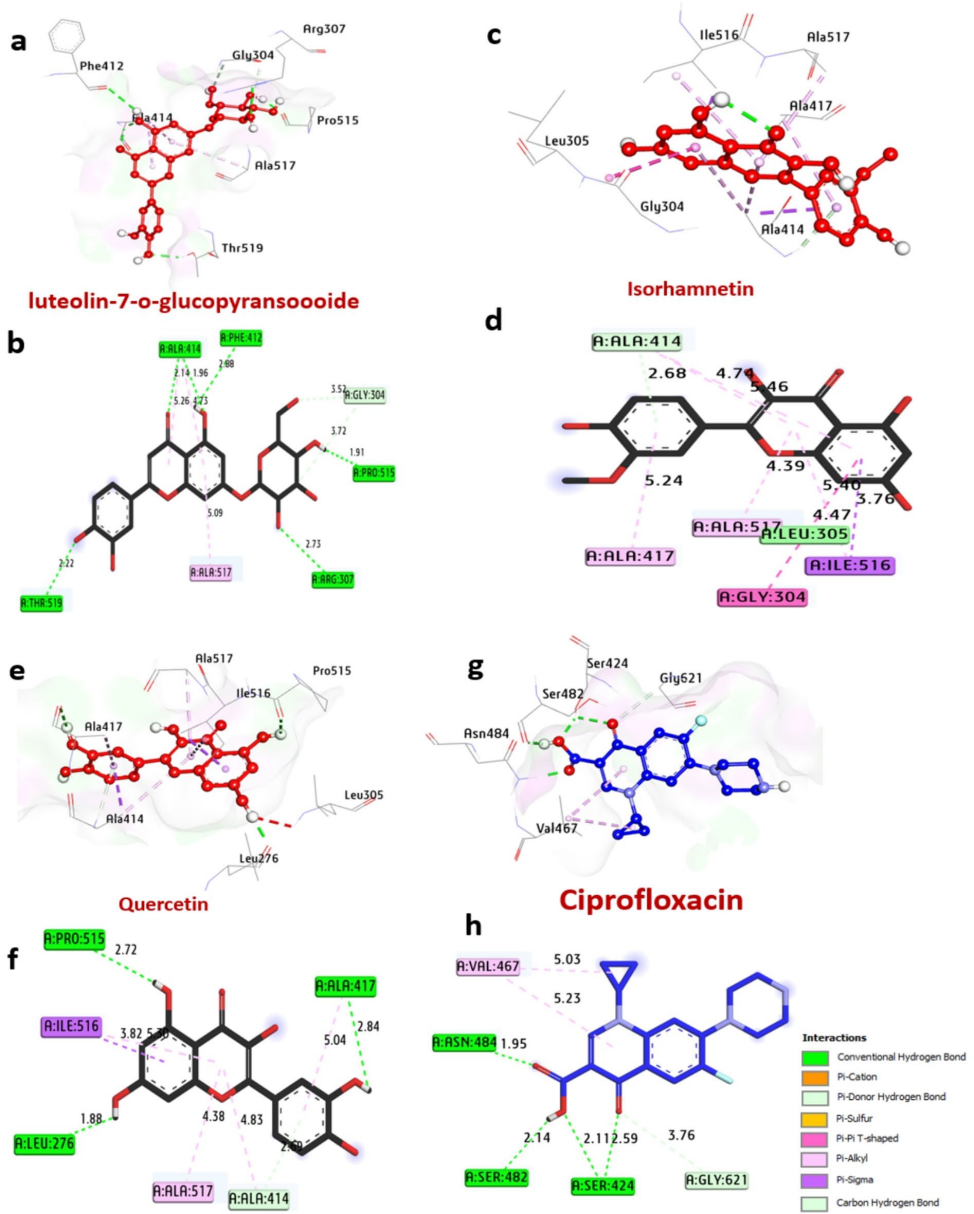
3D representations of compound conformations at the binding pocket of Penicillin-Binding Protein of *E. faecalis* (PDB: ID 6MKI): (**a** and **b**) luteolin 7-*O*-glucopyransoide, (**c** and **d**) Isorhamnetin, (**e** and **f**) Quercetin and (**g** and **h**) Ciprofloxacin.

PBPs are a group of proteins found in the cell membrane of bacteria and are the targets of β-lactam antibiotics like penicillin. These proteins are involved in the final stages of bacterial cell wall synthesis. PBPs play a crucial role in the cell wall synthesis of *E. faecalis*. Among all compounds, the best inhibitors luteolin7-*O*-glucopyransoide, isorhamnetin, and quercetin have the greatest affinity interaction, −7.30, −7.30, and − 7.50 kcal/mol, respectively compared with ciprofloxacin (−6.30 kcal/mol) are shown in Table 9; Fig. 9. Compounds formed hydrogen bonds with the key amino acids: Ala414, Phe412, Thr519, Arg307, Thr519, Pro515, Ala417, and Leu276. Also, several hydrophobic bond interactions within the activity pocket were formed including alkyl bonds with Ala414, (Pi-alkyl) Ala414, Ala517, Ala308, Ala417, Ile516, (Pi-Pi-T shaped) with Gly304, (Carbon H bond) with Ala414 and Gly304, (Pi-Sigma) with Ile516. Furthermore, it can be observed that the amino acids Pro515, Ala414, and Ser424, in the catalytic site enhance the binding affinity.

**Table 9:** Molecular interactions with amino acids of Penicillin-Binding protein of *E. faecalis* (PDB: ID 6MKI).

	Protein	Ligand	3D Structure	Hydrophilic Interactions	Hydrophobic Contacts			No. of H-Bonds	No. of Total Bonds	affinity kcal mol^−1^
				Residue (H- Bond)	Length	Residue (Bond type)	Length			
1	**Penicillin-Binding Protein of** ***E. faecalis*** **(PDB: ID** 6MKI**)**	luteolin7-*O*-glucopyransoide		Ala414, (H- Bond) Phe412, (H- Bond) Pro515, (H- Bond) Thr519, (H- Bond) Arg307, (H- Bond) Ala414, (H- Bond)	2.14 2.88 1.91 2.22 2.73 1.96	Ala414, (Pi-alkyl) Ala414, (Pi-alkyl) Ala517, (Pi- alkyl) Gly304, (Carbon H bond) Gly304, (Carbon H bond)	5.26 4.73 5.09 3.72 3.52	**6**	**11**	**−7.30**
2		Isorhamnetin		-	-	Ala414, (Pi-alkyl) Ala414, (Pi-alkyl) Ala417, (Pi- alkyl) Ala517, (Pi- alkyl) Ile516, (Pi- alkyl) Ile516, (Pi-Sigma) Gly304, (Pi-Pi T shaped)) Ala414, (Carbon H bond)	4.74 5.46 5.24 4.39 5.40 3.76 4.47 2.68	**0**	**9**	**−7.30**
3		Quercetin		Ala417, (H- Bond) Pro515, (H- Bond) Leu276, (H- Bond)	2.84 2.72 1.88	Ala517, (Pi-alkyl) Ala414, (Pi-alkyl) Ala417, (Pi- alkyl) Ile516, (Pi- alkyl) Ile516, (Pi-Sigma) Ala414, (Carbon H bond)	4.38 4.83 5.04 5.30 3.82 2.69	**3**	**9**	**−7.50**
4		**Ciprofloxacin**		Ser424, (H- Bond) Ser424, (H- Bond) Ser482, (H- Bond) Asn484, (H- Bond)	2.59 2.11 2.14 1.95	Val467, (Pi-alkyl) Val467, (Pi-alkyl) Gly621, (Carbon H bond)	5.23 5.03 3.76	**4**	**7**	**−6.30**

#### Docking and interaction with sterol 14-alpha demethylase of *C. albicans*

**Fig. 10:**
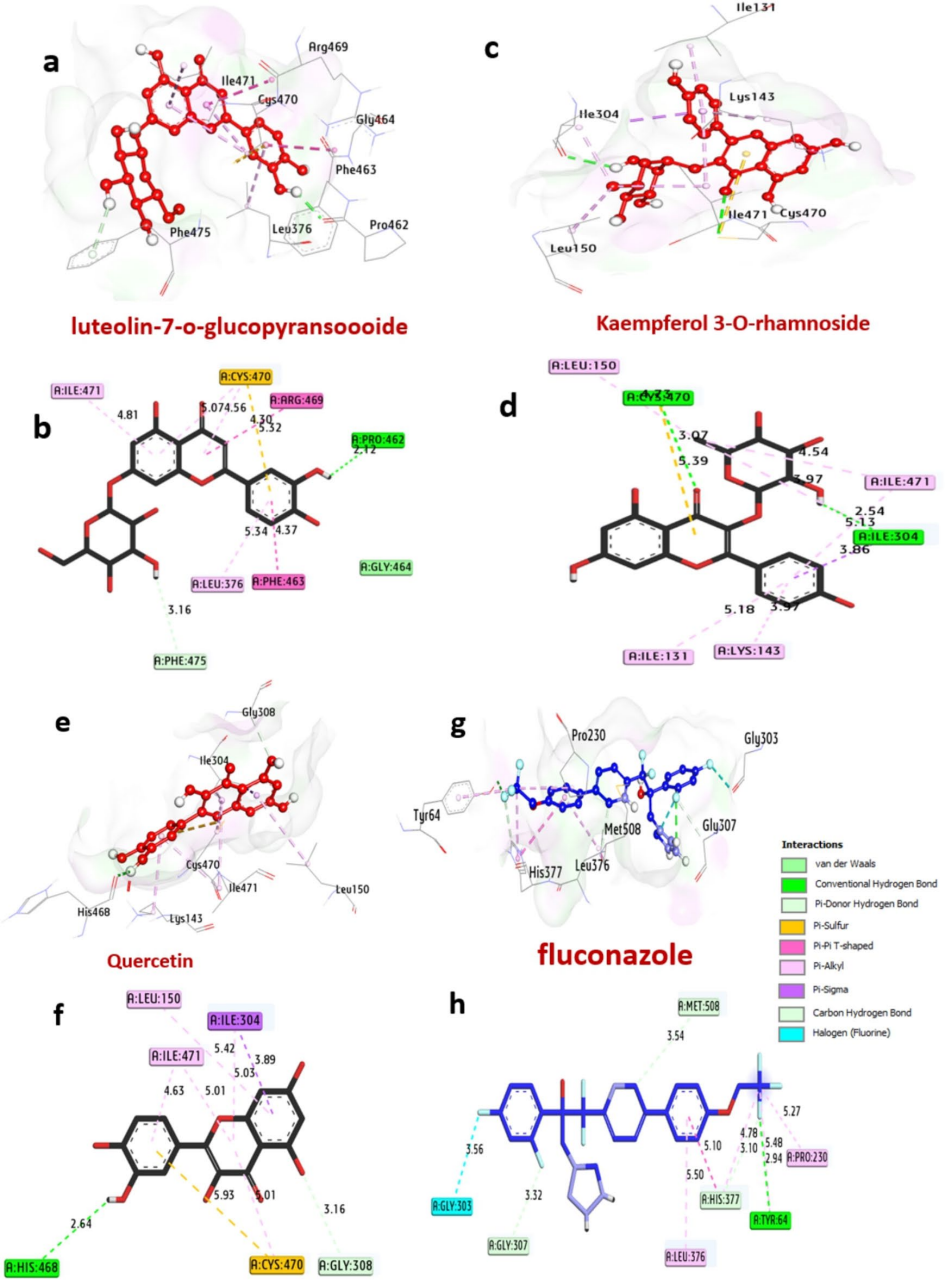
3D representations of compound conformations at the binding pocket of sterol 14-alpha demethylase of *C. albicans* (PDB: ID 5TZ1): (**a** and **b**) luteolin-7-*O*-glucopyransoide, (**c** and **d**) Kaempferol-3-*O*-rhamnoside, (**e** and **f**) Quercetin and (**g** and **h**) fluconazole.

Sterol 14-alpha demethylase is an enzyme that is crucial for the synthesis of ergosterol, an essential component of *C. albicans* cell membrane. Inhibiting this enzyme leads to cell membrane dysfunction and fungal cell death. The docking findings with the selected molecules are shown in Table 10; Fig. 10. Among all compounds, the best inhibitors including luteolin 7-*O*-glucopyransoide, kaempferol 3-*O*-rhamnoside and quercetin have the greatest affinity interaction, −10.20, −9.00 and − 8.80 kcal/mol, respectively compared with fluconazole (−7.20 kcal/mol). Compounds formed hydrogen bonds with the key amino acids: Pro462, Cys470, Arg381, Lys143, Ile304, and His468. Also, several hydrophobic bond interactions within the activity pocket were formed including alkyl bonds with Ile471, Leu376, Cys470, Ala476, Ile379, Leu150, Ile131, Lys143, Ile471, Ile304, (Amid-Pi-stack) with Phe463 and Arg469, (Carbon H bond) with Gly464 and Tyr132, (Pi-Sigma) with Ile304, (Pi-Sulfur) with Ile304 and Cys470, (Carbon H bond) with Gly308. Furthermore, it can be observed that the amino acids Cys470, Arg381, and Ile471, in the catalytic site enhance the binding affinity.

**Table 10:** Molecular interactions of ligands with amino acids of sterol 14-alpha demethylase of *C. albicans* (PDB: ID 5TZ1).

	Protein	Ligand	3D Structure	Hydrophilic Interactions	Hydrophobic Contacts			No. of H-Bonds	No. of Total Bonds	affinity kcal mol^−1^
				Residue (H- Bond)	Length	Residue (Bond type)	Length			
1	**sterol 14-alpha demethylase of** ***C. albicans*** **(PDB: ID 5TZ1)**	luteolin7-*O*-glucopyransoide		Pro462, (H- Bond)	2.12	Ile471, (Pi-alkyl) Leu376, (Pi-alkyl) Cys470, (Pi-alkyl) Cys470, (Pi-alkyl) Phe463 (Amid-Pi-stack) Arg469, (Amid-Pi-stack) Cys470, (Sulfur)	4.81 5.34 5.07 4.56 4.37 5.32	**1**	**8**	**−10.20**
3		Kaempferol3-*O*-rhamnoside		Cys470, (H- Bond) Ile304, (H- Bond)	5.39 5.13	Leu150, (Pi-alkyl) Ile471, (Pi-alkyl) Ile471, (Pi-alkyl) Ile131, (Pi-alkyl) Lys143, (Pi-alkyl) Ile304, (Pi-Sigma) Cys470, (Pi-Sulfur)	4.73 4.54 5.13 5.18 3.97 3.86 5.39	**2**	**10**	**−9.00**
4		Quercetin		His468, (H- Bond)	2.64	Leu150, (Pi-alkyl) Ile471, (Pi-alkyl) Ile471, (Pi-alkyl) Ile304, (Pi-alkyl) Cys470, (Pi-alkyl) Ile304, (Pi-Sigma) Cys470, (Pi-Sulfur) Gly308, (Carbon H bond)	5.42 4.63 5.01 5.03 5.01 3.98 5.93 3.16	**1**	**9**	**−8.80**
5		**Fuconazole**		Tyr64, (H- Bond)	2.94	Pro230, (Pi-alkyl) Tyr64, (Pi-alkyl) His377, (Pi-alkyl) Leu376, (Pi-alkyl) Gly303, (Halogen) His377, (Carbon H bond) Met508, (Carbon H bond) Gly307, (Carbon H bond)	5.27 5.48 4.78 5.50 3.56 3.10 3.32 3.54	**1**	**9**	**−7.20**

### In Silico pharmacokinetics ADME prediction of synthesized compounds

**Fig. 11:**
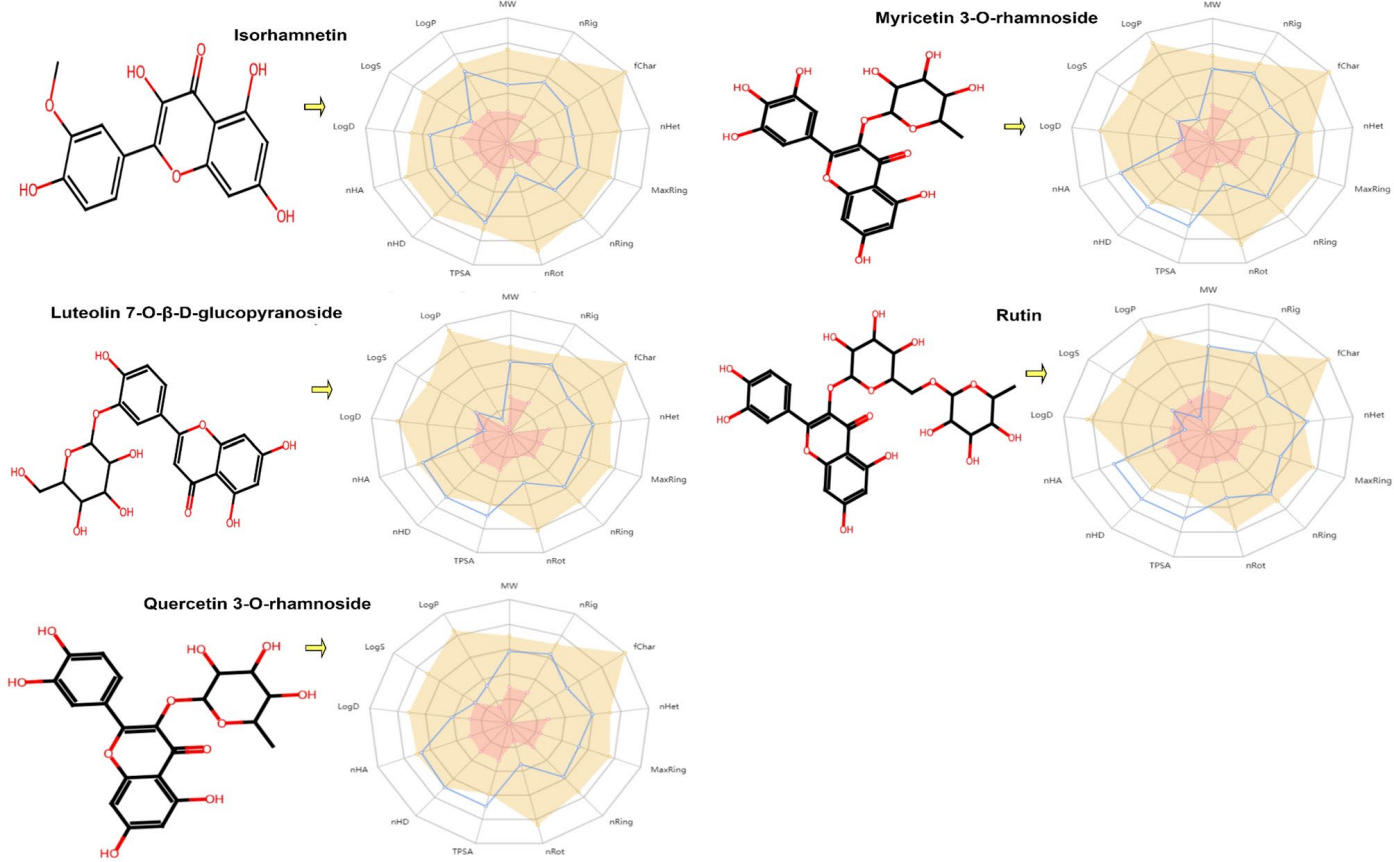
Oral bio-availability graph for compounds.

Based on the docking results, the physiochemical properties of the promising compounds are shown in Table 11; Fig. 11. All physiochemical criteria were examined and evaluated. Therefore, all the compounds have molecular weight below 500 except rutin and possess enough rotatable bonds (RBs-6), crucial for high structural flexibility. This is important because compounds with less than ten RBs are more likely to be bioavailable. As RBs increase, they become more critical in determining a successful interaction with certain binding sites. It was found that all five compounds had less than 10 HBA and less than 5 HBD, indicating a favorable balance of HBA and HBD and a higher likelihood of oral bioavailability. Additionally, The TPSA values of the compounds were found to be relatively moderate, with most falling in the optimal range of 120.0-269.0 for good absorption in the gut and oral bioavailability. Additionally, the evaluation included an assessment of the lipophilicity and water solubility of the compounds. The results revealed that all active compounds exhibit high solubility in water. Their Log S values fall within the range of −3.70 to −2.40, signifying a high degree of water solubility. The presence of such soluble molecules simplifies the synthesis, handling, and formulation of bioactive substances. Subsequently, the compounds underwent pharmacokinetic testing. The findings indicate that the compounds under investigation have a high theoretical bioavailability, making them potential drug-like agents. However, all compounds had moderate intestinal absorption. These chemicals may also interact with other medicines since they inhibit the enzymes CYP2C9, and CYP1A2. The study proceeded to evaluate the drug-likeness of the compounds utilizing Lipinski, Golden Triangle, and Pfizer rules. It was observed that all compounds met the drug-likeness criteria outlined by Lipinski and the Golden Triangle rules, indicating that they possess favorable physicochemical properties for drug development. Furthermore, the distribution of compounds, including Plasma Protein Binding (PPB), was scrutinized. It was found that all compounds exhibited PPB values higher than 83.81%, indicating a high degree of protein binding in plasma, a low therapeutic index, and a minimal fraction of unbound plasma. Additionally, the Blood-Brain Barrier (BBB) penetration analysis suggested that all compounds were classified as BBB-, signifying their inability to traverse the blood-brain barrier. Conclusively, based on computational assessments, compounds Quercetin 3-*O*-rhamnoside and Kaempferol 3-*O*-rhamnoside were deemed relatively safe and non-toxic according to the data presented in Table 12.

**Table 11:** Prediction of pharmacokinetics and physicochemical properties of compounds.

Id	ID	Isorhamnetin	Myricetin 3-O-rhamnoside	Luteolin 7-O-β-D-glucopyranoside	Rutin	Quercetin 3-O-rhamnoside	Id	ID	Isorhamnetin	Myricetin 3-O-rhamnoside	Luteolin 7-O-β-D-glucopyranoside	Rutin	Quercetin 3-O-rhamnoside
**Physicochemical Properties**	MW	**316.06**	**464.1**	**448.1**	**610.15**	**448.1**	**Metabolism**	**CYP1A2-inh**	**0.96**	**0.079**	**0.087**	**0.013**	**0.098**
	Vol	**300.063**	**421.937**	**413.147**	**552.318**	**413.147**		**CYP1A2-sub**	**0.736**	**0.063**	**0.05**	**0.026**	**0.064**
	Dense	**1.053**	**1.1**	**1.085**	**1.105**	**1.085**		**CYP2C19-inh**	**0.111**	**0.014**	**0.017**	**0.011**	**0.019**
	nHA	**7**	**12**	**11**	**16**	**11**		**CYP2C19-sub**	**0.049**	**0.046**	**0.054**	**0.05**	**0.05**
	nHD	**4**	**8**	**7**	**10**	**7**		**CYP2C9-inh**	**0.657**	**0.031**	**0.01**	**0.002**	**0.043**
	TPSA	**120.36**	**210.51**	**190.28**	**269.43**	**190.28**		**CYP2C9-sub**	**0.827**	**0.218**	**0.381**	**0.246**	**0.5**
	nRot	**2**	**3**	**4**	**6**	**3**		**CYP2D6-inh**	**0.585**	**0.038**	**0.056**	**0.007**	**0.141**
	nRing	**3**	**4**	**4**	**5**	**4**		**CYP2D6-sub**	**0.304**	**0.164**	**0.209**	**0.155**	**0.194**
	MaxRing	**10**	**10**	**10**	**10**	**10**		**CYP3A4-inh**	**0.52**	**0.043**	**0.079**	**0.013**	**0.075**
	nHet	**7**	**12**	**11**	**16**	**11**		**CYP3A4-sub**	**0.086**	**0.011**	**0.019**	**0.003**	**0.018**
	fear	**0**	**0**	**0**	**0**	**0**	**Excretion**	**CL (Clearance)**	**6.991**	**5.262**	**4.051**	**1.349**	**6.373**
	nRig	**18**	**24**	**24**	**30**	**24**		**T12**	**0.922**	**0.873**	**0.676**	**0.524**	**0.8**
	Flex	**0.111**	**0.125**	**0.167**	**0.2**	**0.125**	**Toxicity**	**hERG Blockers**	**0.061**	**0.015**	**0.034**	**0.017**	**0.011**
	nStereo	**0**	**5**	**5**	**10**	**5**		**H-HT**	**0.064**	**0.164**	**0.098**	**0.092**	**0.137**
**Solubility**	LogS	**−3.748**	**−3.962**	**−3.839**	**−3.928**	**−4.03**		**DILI**	**0.978**	**0.982**	**0.92**	**0.982**	**0.98**
	LogD	**2.262**	**0.909**	**0.819**	**0.695**	**1.594**		**AMES Toxicity**	**0.596**	**0.728**	**0.695**	**0.805**	**0.82**
	LogP	**2.541**	**0.438**	**0.234**	**−0.763**	**0.819**		**Rat OralToxicity**	**0.074**	**0.063**	**0.028**	**0.05**	**0.094**
	ESOL Log S	−6.005	−6.986	−6.776	−5.943	−6.107		**FDAMDD**	**0.462**	**0.234**	**0.029**	**0.014**	**0.135**
	Ali Log S	−6.005	−6.986	−6.776	−5.943	−6.107		**Skin Sensitization**	**0.774**	**0.603**	**0.176**	**0.036**	**0.181**
	Silicon-IT class	Soluble	Soluble	Soluble	Soluble	Soluble		**Carcinogenicity**	**0.047**	**0.034**	**0.113**	**0.064**	**0.068**
**drug-likeness**	**Lipinski Rule**	Accepted	Rejected	Rejected	Rejected	Rejected		**Eye Corrosion**	**0.007**	**0.003**	**0.003**	**0.003**	**0.003**
	**Pfizer Rule**	Accepted	Accepted	Accepted	Accepted	Accepted		**Eye Irritation**	**0.919**	**0.171**	**0.017**	**0.01**	**0.173**
	**Golden Triangle**	Accepted	Accepted	Accepted	Rejected	Accepted		**Respiratory Toxicity**	**0.121**	**0.047**	**0.045**	**0.015**	**0.041**
**Absorption**	Pgp-inh	0.008	0.003	0.001	0.002	0.003	**Toxicophoric Rules**	**N.G. Carcinogenicity**	**0**	**0**	**0**	**0**	**0**
	Pgp-sub	0.042	0.584	0.912	0.978	0.607		**LD50_oral**	**0**	**1**	**0**	**0**	**0**
	HIA	0.024	0.698	0.848	0.925	0.534		**G. Carcinogenicity**	**0**	**0**	**0**	**0**	**0**
	F (20%)	0.03	0.09	0.937	0.234	0.033		**Sure ChEMBL**	**8**	**8**	**7**	**8**	**8**
	F (30%)	0.978	0.997	0.999	0.999	0.994		**Non-Biodegradable**	**0**	**2**	**2**	**2**	**2**
	Caco-2	−5.056	−6.267	−6.179	−6.336	−6.142		**Skin Sensitization**	**1**	**2**	**1**	**2**	**2**
	MDCK	9.45E-06	6.64E-06	2.75E-05	2.97E-05	6.55E-06		**Aquatic Toxicity**	**0**	**0**	**0**	**0**	**0**
**Distribution**	BBB	0.005	0.009	0.078	0.111	0.012	**Medicinal Chemistry**	**Toxicophores**	**0**	**1**	**0**	**0**	**0**
	PPB%	96.23%	87.71%	87.33%	83.81%	89.52%		**QED**	**0.572**	**0.244**	**0.279**	**0.14**	**0.276**
	VDss	0.647	0.903	0.884	0.754	0.839		**Synth**	**2.453**	**4.149**	**3.934**	**4.783**	**4.008**
	Fu %	8.51%	14.29%	13.70%	20.87%	11.10%		**Fsp3**	**0.062**	**0.286**	**0.286**	**0.444**	**0.286**

**Table 12:** Prediction of toxicity risks and oral toxicity prediction results of compounds.

No	Ligand	Toxicity risks				Physicochemical properties					
		Mutagenic	Tumorigenic	Irritant	Reproductive	CLogP	Solubility	Molecular Weight	TPSA	Drug likeness	Drug score
1	**Isorhamnetin**	(-)	(-)	(-)	(-)	1.77	−2.80	316.0	116.4	1.17	0.47
2	**Kaempferol 3**,**7-*O*-dirhamnoside**	(+)	(-)	(-)	(+)	0.14	−3.70	592.0	214.0	0.18	0.15
3	**Kaempferol 3-** ***O*** **-rhamnoside**	(-)	(-)	(-)	(-)	0.93	−2.99	432.0	166.0	2.80	0.77
4	**Myricetin 3-** ***O*** **-rhamnoside**	(+)	(+)	(-)	(-)	1.49	−2.49	302.0	127.4	1.60	0.30
5	**Quercetin 3-** ***O*** **-rhamnoside**	(-)	(-)	(-)	(-)	0.58	−2.70	448.0	186.3	3.31	0.77
6	**Quercetin**	(+)	(+)	(-)	(-)	1.49	−2.49	302.0	127.4	1.60	0.30
7	**Rutin**	(-)	(-)	(-)	(-)	−1.26	−2.40	610.0	265.0	3.31	0.57
8	**Luteolin 7-** ***O*** **-glucopyransoide**	(-)	(-)	(-)	(-)	0.00	−2.45	448.0	186.3	−3.43	0.41

### Molecular dynamics simulation (MDS)

**Fig. 12:**
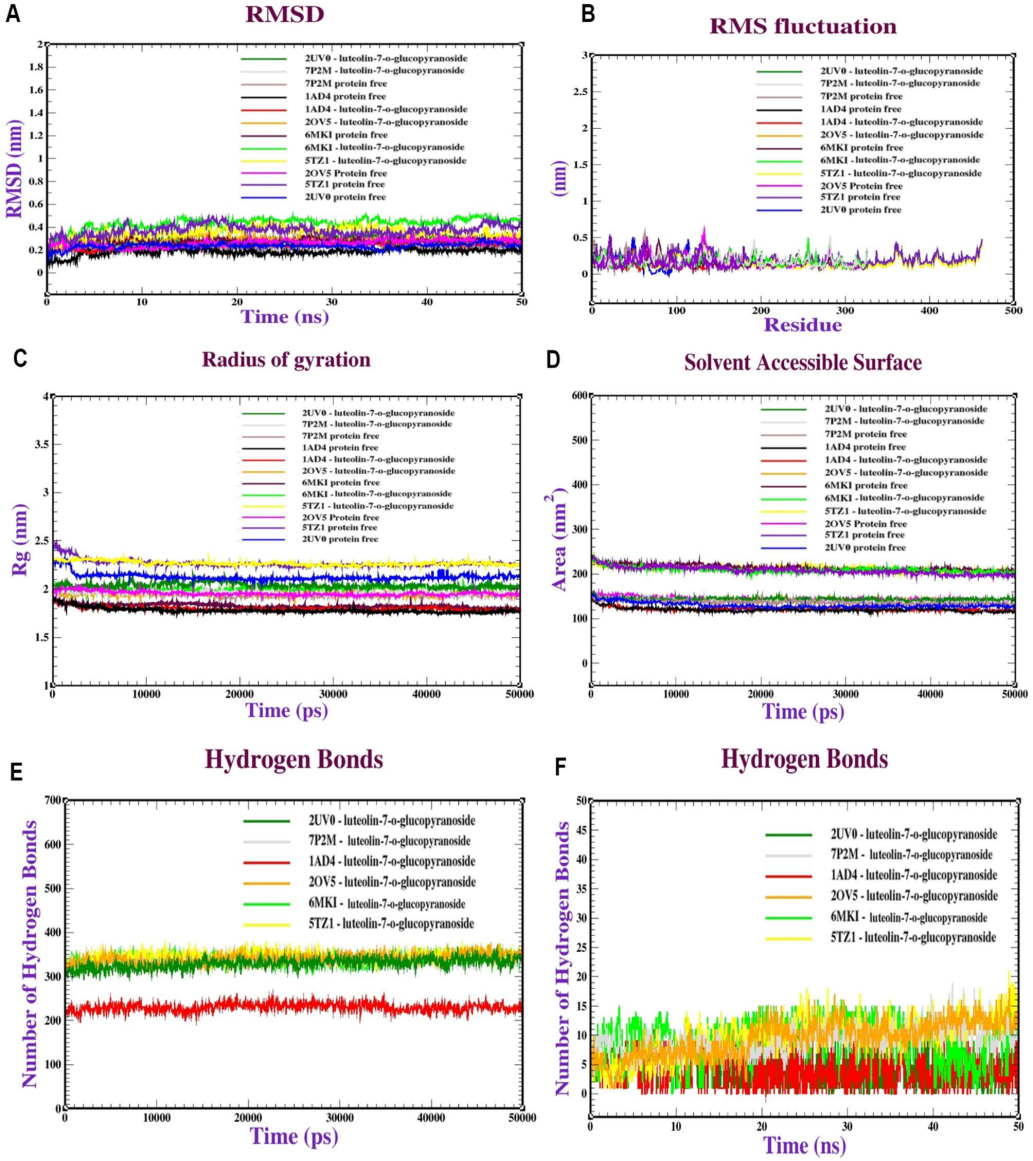
Molecular dynamics of six anti-microbial activity receptors including (1AD4, 2UV0, 2OV5, and 5TZ1) comlexed with luteolin-7-*O-*glucopyransoide: (A) RMSD, (B) RMSF, (C) SASA, (D) Radius of gyration (Rg), (E) Intramolecular hydrogen bonds and (F) Intermolecular hydrogen bonds.

After analyzing the binding of six anti-microbial activity receptors with a promising luteolin7-*O*-glucopyransoide, dynamic simulations were conducted to study the behavior and stability of the protein complex at the atomic level. **Firstly**, several analyses of the MDS of dihydropteroate synthase of *S. aureus* (PDB: ID 1AD4), LasR protein in *P. aeruginosa* (PDB: ID 2UV0), KPC-2 carbapenemase of *K. pneumoniae* (PDB: ID 2OV5), and sterol 14-alpha demethylase of *C. albicans* (PDB: ID 5TZ1) were complexed with luteolin-7-*O*-glucopyransoide performed to assess the stability and dynamics of the complexes. The Root Mean Square Deviation (RMSD) was utilized to evaluate the stability of the protein structures. **(**Fig. 12:**A)**. The RMSD values for 1AD4, 2UV0, 2OV5, and 5TZ1 proteins with luteolin-7-o-glucopyransoide remained stable, ranging from 0.18 to 0.20 nm, 0.22–0.25 nm, 0.25–0.30 nm, and 0.25–0.40 nm, respectively, stabilizing after 20, 25, and 30 ns. Similarly, the RMSD values for DNA Gyrase of E.coli (PDB: ID 7P2M) and Penicillin-Binding Protein of E. faecalis (PDB: ID 6MKI) proteins with luteolin7-O-glucopyransoide were stable, ranging from 0.20 to 0.22 nm and 0.35–0.45 nm, stabilizing after 20 and 15 ns, respectively. **Secondly**, the flexibility of amino acid residues during the simulation was evaluated using Root Mean Square Fluctuation (RMSF) analysis, indicating minor variability with most residues showing minimal fluctuations (0.1–0.6 nm), suggesting relative stability for antimicrobial receptors like 1AD4, 2UV0, 2OV5, 6MKI, 7P2M, and 5TZ1 proteins. **(**Fig. 12:**B)**. **Thirdly**, Radius of Gyration (Rg) analysis was conducted to assess the overall shape of the protein complexes, with Rg values indicating the compactness or expansion of the protein structures during the simulation. Figure 12:**C** shows Rg values of 1AD4, 2UV0, 2OV5, and 5TZ1 protein with luteolin-7-o-glucopyransoide complexes ranging from 1.85 to 1.90 nm, 2.15–2.20 nm, 1.95–2.10 nm, and 2.30–2.50 nm, respectively. Additionally, Rg values of 7P2M and 6MKI proteins with luteolin7-O-glucopyransoide were stable ranging from 2.00 to 2.05, and 2.10–2.15 nm, respectively. **Fourthly**, SASA used to assess the exposure of protein to the surrounding solvent molecules. It calculates the surface area of a protein that is accessible to solvent molecules, which can provide insights into the protein folding, dynamics, and interactions with molecules. Figure 12:**D** shows SASA values of 1AD4, 2UV0, 2OV5, and 5TZ1 proteins with luteolin7-*O*-glucopyransoide complexes ranging from 135 to 145, 150–160, 155–165, and 220–235 nm^2^, respectively. Additionally, SASA values of 7P2M and 6MKI proteins with luteolin7-O-glucopyransoide were stable ranging from 2.00 to 2.05, and 2.10–2.15 nm^2^, respectively. **Finally**, intramolecular hydrogen bonds play a pivotal role in influencing their characteristics, affecting attributes such as structure, stability, and reactivity. Within the context of the analysis, Fig. 12:**E** illustrates the presence of intramolecular hydrogen bonds in the complexes involving proteins 1AD4, 2UV0, 2OV5, and 5TZ1 with luteolin7-*O*-glucopyransoide, showcasing bond strengths ranging from 205 to 240, 310–340, 360–380, and 350–380 nm², respectively. Similarly, proteins 7P2M and 6MKI in conjunction with luteolin7-*O*-glucopyransoide exhibit bond strengths within the range of 320–350 nm² and 320–360 nm², respectively. Concerning intermolecular hydrogen bonds (Fig. 12:**F)**, the majority of protein receptor complexes display a significant number of interactions, ranging from 2 to 14 bonds. These intermolecular hydrogen bonds play a crucial role in bolstering the stability of the intricate structures formed by these complexes.

## Discussions

In the last decade, there has been a concentrated effort in research to identify natural alternatives to synthetic food preservatives and disinfectants^49^. The emergence of new natural antimicrobial extracts and molecules is projected to improve the preservation of raw and minimally processed foods, which will help retain their organoleptic and nutritional attributes, as well as decrease food waste by extending the shelf life of perishable products^50^. In this regard, extracts derived from plants are considered promising candidates for antimicrobial substances, particularly phenolic compounds like flavonoids and phenolic acids. These compounds are known for their diverse biological activities, which encompass antioxidant and antimicrobial properties^51^^,^^52^.

The current investigation focuses on the phenolic and flavonoid constituents of the aerial parts of *P. anisum* and *C. sativum*, with particular attention given to the extraction of the major flavonoids present in these plant parts. Moreover, the study analyzed the antimicrobial properties these two plant extracts at different concentrations (2.0, 1.0, and 0.5 mg/mL) against a variety of microorganisms.

In comparison to earlier studies, the antimicrobial effectiveness of *P. anisum* and *C. sativum* is consistent with established research regarding the function of phenolic compounds in plant defense systems. Research has repeatedly shown that phenolics, which include flavonoids, tannins, and phenolic acids, play a significant role in antimicrobial activity by damaging microbial structures and hindering enzymatic processes^53^. Previous investigations have revealed that *P. anisum* exhibits potent antimicrobial properties, largely due to anethole, a phenolic compound present in substantial amounts^54^. Likewise, *C. sativum* has been recognized as a potent antimicrobial agent owing to its phenolic components, such as caffeic and chlorogenic acids, which demonstrate significant inhibitory effects against both bacteria and fungi^55^^,^^56^.

Compared to conventional antimicrobial agents sourced from plants, *P. anisum* and *C. sativum* exhibit a wider range of antimicrobial effectiveness, especially against foodborne pathogens and strains resistant to antibiotics. Previous literature reviews highlight the significant role of phenolic compounds in natural antimicrobial formulations, positioning these plants as viable alternatives in pharmaceutical and food preservation sectors. Nonetheless, certain studies indicate that their effectiveness may fluctuate based on extraction techniques, the maturity of the plants, and environmental factors that affect phenolic content^9^^,^^17^.

The findings of this research indicate that methanol extracts obtained from the aerial parts of *P. anisum* and *C. sativum* displayed significantly potent antimicrobial activities against a range of gram-negative and gram-positive bacteria, as well as the yeast *Candida albicans*. The differing percentages of inhibition observed may be linked to the variations in their phytochemical constituents **[57; 58]**. The results obtained in this study are consistent with those reported by **Oulahal and Degraeve**^59^, **Hemdan et al.**^60^, and **Paunova-Krasteva et al.**^61^, who proposed a positive link between the total phenolic content and the antimicrobial effectiveness of the plant extracts.

Various mechanisms through which plant phenolics exert their antimicrobial effects against bacteria and yeasts have been documented. The most frequently observed mechanisms occur at the membrane level^62^. Several authors have reported that these mechanisms can lead to dose-dependent modifications in microbial membranes, which may include reversible permeability changes as well as complete membrane disruption, causing the release of intracellular materials^63^. A commonly suggested mechanism involves the hydroxyl (-OH) groups present in the phenolic structure, which promote interactions through hydrogen bonding with the microbial cell envelope. Furthermore, phenolic compounds have the capacity to accumulate on the surface of the cell envelope, allowing them to penetrate or even traverse the membrane and enter the cytoplasm of microbial cells. Once inside, these compounds can interact with a range of cellular constituents or modify the pH levels within the cell. The consequences of phenolic infiltration into the cytoplasm are significant, leading to disruptions in DNA and RNA synthesis, interference with protein synthesis and functionality, and alterations in intermediary metabolic processes, particularly those related to ATP generation. Furthermore, phenolics can affect membrane proteins that are crucial for various cellular functions, including the inhibition of proteins that facilitate bacterial cell division, thus hindering the initial phase of this process^64^. In this scenario, Gram-negative bacteria, characterized by their hydrophilic cell wall, exhibit reduced sensitivity to the hydrophobic compounds of polyphenols compared to their Gram-positive bacteria^65^. These findings, along with the varying susceptibility of Gram-negative and Gram-positive bacteria to phenolic acids, reinforce the notion that the structural and compositional differences in cell membranes are crucial in determining the susceptibility to plant phenolics^66^. Our findings suggest that the methanol extracts of anise and coriander aerial parts containing phenolic compounds possess promising antimicrobial properties against the studied pathogens. Similar studies have also supported the antimicrobial effectiveness against pathogenic bacteria^31^. Furthermore, related research employed pine needle leaf extract to evaluate antibacterial activity against six uropathogenic bacteria (*S. aureus*,* S. haemolyticus*,* E. faecalis*,* E. coli*,* K. pneumoniae*, and *P. aeruginosa*)^67^.

**Fig. 13:**
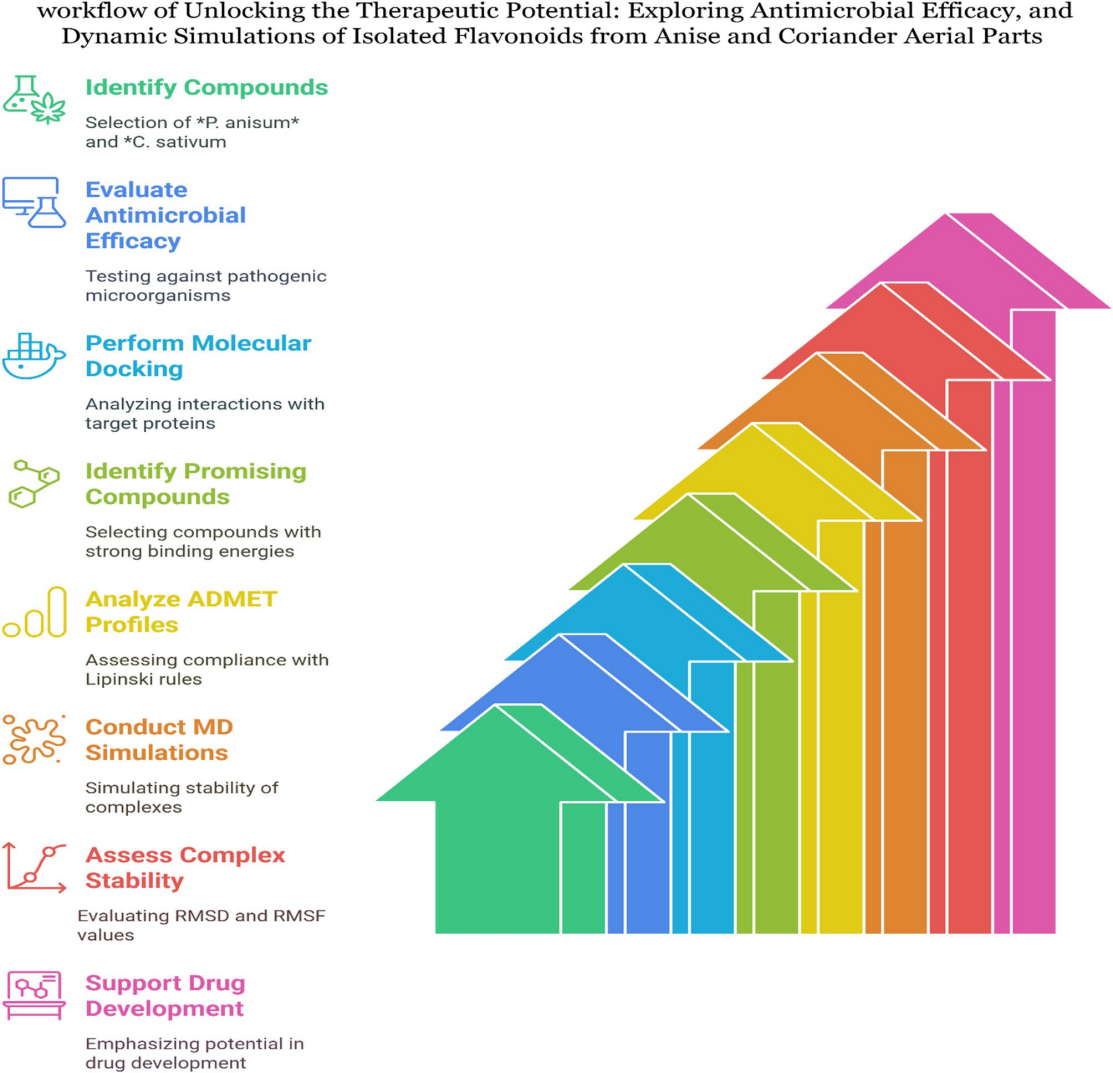
workflow of unlocking the Therapeutic Potential: Exploring Antimicrobial Efficacy, and Dynamic Simulations of Isolated Flavonoids from *Anise and Coriander* Aerial Parts.

Computational analysis used to elucidate the relationship between isolated phytochemicals and their antimicrobial effects through various computational analyses, including molecular docking, pharmacokinetics, and molecular dynamics simulations^68^. The compounds likely inhibit the dihydropteroate synthase enzyme in *S. aureus*, consistent with findings by **Khidre** et al.^69^. Key residues Thr75, Thr115, and Arg61 at the catalytic site of the LasR protein in *P. aeruginosa* were found to enhance binding affinity, aligning with^19^. Isorhamnetin and quercetin exhibited the highest affinity (−8.10 kcal/mol) against DNA Gyrase of *E. coli*, supported by **Sroor** et al.^70^ who performed antimicrobial activity of identified compounds against *E.coli* and then validated their inhibition using in silico interaction between isolated ligands and DNA Gyrase. Luteolin 7-*O*-glucopyranoside showed the greatest affinity (−9.60 kcal/mol), with specific residues Ser130, Thr235, and Ser70 enhancing binding, corroborated by **Mukhtar** et al.^71^. These findings, along with demonstrated in vitro antibacterial activity, suggest that these compounds could serve as potent bacterial inhibitors, consistent with **Sroor** et al.^70^ who used docking studies to explain the potential antimicrobial effects, of key enzymes such as Sterol 14-demethylase of *C. albicans*. Pharmacokinetics study evaluated several compounds, revealing that most exhibited favorable physicochemical properties for drug development, such as molecular weights below 500 and good oral bioavailability. The compounds demonstrated high water solubility and theoretical bioavailability, though they had moderate intestinal absorption. They met drug-likeness criteria and showed high plasma protein binding, indicating a low therapeutic index. However, they were unable to cross the blood-brain barrier. Quercetin 3-*O*-rhamnoside, and Kaempferol 3-*O*-rhamnoside were considered relatively safe and non-toxic. Similar findings were reported by **Melk & El-Sayed**^72^, who tested the pharmacokinetics of compounds identified from plant extracts with biological activity. Molecular Dynamics simulations were conducted to confirm the stability of complexes formed by dihydropteroate synthase of *S. aureus*, LasR protein in *P. aeruginosa*, KPC-2 carbapenemase of *K. pneumoniae*, and sterol 14-alpha demethylase of *C. albicans* in association with luteolin7-*O*-glucopyransoide. These simulations aimed to evaluate the stability and dynamics of the complexes. The stability was evidenced by RMSD values ranging from 0.18 to 0.40 nm, indicating minimal fluctuations in Root Mean Square Fluctuation (RMSF) values, which ranged from 0.10 to 0.60 nm. The SASA values fell within the range of 135 to 235 nm², while the Radius of Gyration (Rg) values varied from 1.85 to 2.50 nm, offering insights into the structural shapes of the protein complexes. These findings were in line with the research conducted by **Melk & El-Sayed**^72^, which also employed dynamics simulations to explore the stability and conformational intricacies of proteins in conjunction with promising compounds. Figures (13) illustrated workflow of unlocking the therapeutic potential: exploring antimicrobial efficacy, and dynamic simulations of isolated flavonoids from anise and coriander aerial parts.

## Conclusion

The study evaluated the antimicrobial efficacy of methanol extract of *P. anisum* and *C. sativum* aerial parts against pathogenic microorganisms. Subsequently, molecular docking was employed to analyze interactions between promising compounds and antimicrobial target proteins. Compounds such as luteolin7-*O*-glucopyranoside, isorhamnetin, and quercetin demonstrated strong binding energies, effectively interacting with the active sites of antimicrobial protein receptors. These interactions suggest potential for enzyme inhibition and significant antimicrobial effects. Additionally, in-silico ADMET profiles indicated compliance with Lipinski rules, reflecting favorable physicochemical properties. MD simulations revealed stable complexes between luteolin7-*O*-glucopyranoside and antimicrobial receptors (1AD4, 2UV0, 2OV5, and 5TZ1). The stability of these complexes was supported by RMSD values (0.18 to 0.40 nm) and minor fluctuations in RMSF values (0.10 to 0.60 nm). SASA values ranged from 135 to 235 nm², and Rg values varied from 1.85 to 2.50 nm, providing insights into the shapes of the protein complexes. These findings bolster the compounds’ potential in ongoing drug development endeavors, emphasizing their stability and suitability for further exploration in drug development processes.

## Electronic supplementary material

Below is the link to the electronic supplementary material.

Supplementary Material 1

## Data Availability

All data produced or examined in the course of this research has been embedded within the content of this published article.
