# Supplementary material for: Therapeutic potential of isolated flavonoids from Anise and coriander aerial parts in antimicrobial efficacy, molecular docking, ADMET, and dynamic simulations
Source: Sci Rep. 2025 Jul 21;15:26485. doi: 10.1038/s41598-025-10927-w (PMC12280138; doi:10.1038/s41598-025-10927-w)
Supplement: Supplementary file 1 — Supplementary Material 1 [file 41598_2025_10927_MOESM1_ESM.docx]

**Table S1:** Antibacterial activities of the most active extract by agar diffusion method.

| **No** | **Extract** | **Conc.**  **mg/mL** |  | Antibacterial activities (mm) | | | | |
| --- | --- | --- | --- | --- | --- | --- | --- | --- |
|  |  |  | ***E. coli***  ATCC25915  (G - ve) | ***S. aureus***  ATCC25923  (G + ve) | ***E. faecalis***  ATCC29212  (G + ve) | ***P. aeruginosa***  ATCC10145  (G -ve) | ***K. pneumoniae*** ATCC 25175  (G -ve) | *C. albicans* |
| 1 | **DMSO** | - | (-) | (-) | (-) | (-) | (-) | (-) |
| 2 |  | 2.0 | 7.00 ± 0.05 | 8.50 ± 0.09 | 3.50 ± 0.08 | 6.80 ± 0.01 | 10.0 ± 0.10 | 6.00 ± 0.00 |
| 3 | **A** | 1.0 | 3.50 ± 0.00 | 4.00 ± 0.10 | (-) | 3.50 ± 0.00 | 7.50 ± 0.08 | 3.50 ± 0.00 |
| 4 |  | 0.5 | 3.00 ± 0.01 | 2.00 ± 0.00 | (-) | 2.00 ± 0.00 | 4.00 ± 0.00 | 3.00 ± 0.01 |
| 5 |  | 2.0 | 6.55 ± 0.08 | 9.00 ± 0.20 | 4.50 ± 0.07 | 7.00 ± 0.10 | 8.55 ± 0.00 | 8.00 ± 0.20 |
| 6 | **B** | 1.0 | 2.50 ± 0.00 | 4.50 ± 0.04 | (-) | 3.00 ± 0.00 | 5.50 ± 0.10 | 5.60 ± 0.00 |
| 7 |  | 0.5 | 2.00 ± 0.00 | 3.00 ± 0.10 | (-) | 2.00 ± 0.08 | 2.00 ± 0.10 | 2.00 ± 0.06 |
| 8 | **Ciprofloxacin** | 50 µg.mL | 10.5 ± 0.00 | 8.40 ± 0.10 | 6.30 ± 0.00 | 9.50 ± 0.10 | 11.00 ± 0.00 |  |
| Values are given as mean ± standard error.  (G + ve) Gram-Positive, (G - ve) Gram-Negative, (-) Negative.  **(A)** for methanol extract of *P. anisum* aerial parts and **(B)** for methanol extract of *C. sativum* aerial parts. | | | | | | | | |

**Table S2:** MIC of the most active extract by agar diffusion method.

| **Extract** |  | MIC mg.mL | | | | | |
| --- | --- | --- | --- | --- | --- | --- | --- |
|  | ***E. coli***  ATCC25915  (G - ve) | ***S. aureus***  ATCC25923  (G + ve) | ***E. faecalis***  ATCC29212  (G + ve) | ***P. aeruginosa***  ATCC10145  (G -ve) | ***K. pneumoniae*** ATCC 25175  (G -ve) | *C. albicans* | |
| **A** | 0.25 | 0.25 | 2.0 | 0.25 | 0.25 | 0.25 | |
| **B** | 0.25 | 0.25 | 2.0 | 0.25 | 0.25 | 0.25 | |
| Values are given as mean ± standard error.  (G + ve) Gram-Positive, (G - ve) Gram-Negative, (-) Negative.  **(A)** for methanol extract of *P. anisum* aerial parts and **(B)** for methanol extract of *C. sativum* aerial parts. | | | | | | |  |
